# Predicting the potential for zoonotic transmission and host associations for novel viruses

**DOI:** 10.1038/s42003-022-03797-9

**Published:** 2022-08-19

**Authors:** Pranav S. Pandit, Simon J. Anthony, Tracey Goldstein, Kevin J. Olival, Megan M. Doyle, Nicole R. Gardner, Brian Bird, Woutrina Smith, David Wolking, Kirsten Gilardi, Corina Monagin, Terra Kelly, Marcela M. Uhart, Jonathan H. Epstein, Catherine Machalaba, Melinda K. Rostal, Patrick Dawson, Emily Hagan, Ava Sullivan, Hongying Li, Aleksei A. Chmura, Alice Latinne, Christian Lange, Tammie O’Rourke, Sarah Olson, Lucy Keatts, A. Patricia Mendoza, Alberto Perez, Cátia Dejuste de Paula, Dawn Zimmerman, Marc Valitutto, Matthew LeBreton, David McIver, Ariful Islam, Veasna Duong, Moctar Mouiche, Zhengli Shi, Prime Mulembakani, Charles Kumakamba, Mohamed Ali, Nigatu Kebede, Ubald Tamoufe, Samuel Bel-Nono, Alpha Camara, Joko Pamungkas, Kalpy J. Coulibaly, Ehab Abu-Basha, Joseph Kamau, Soubanh Silithammavong, James Desmond, Tom Hughes, Enkhtuvshin Shiilegdamba, Ohnmar Aung, Dibesh Karmacharya, Julius Nziza, Daouda Ndiaye, Aiah Gbakima, Zikankuba Sajali, Supaporn Wacharapluesadee, Erika Alandia Robles, Benard Ssebide, Gerardo Suzán, Luis F. Aguirre, Monica R. Solorio, Tapan N. Dhole, Nguyen T. T. Nga, Peta L. Hitchens, Damien O. Joly, Karen Saylors, Amanda Fine, Suzan Murray, William B. Karesh, Peter Daszak, Jonna A. K. Mazet, PREDICT Consortium, Cátia Dejuste de Paula, Christine K. Johnson

**Affiliations:** 1 One Health Institute, School of Veterinary Medicine, University of California, Davis, Davis, CA 95616 USA; 2 Center for Infection and Immunity, Columbia University, New York, NY 10032 USA; 3 EcoHealth Alliance, 520 Eighth Avenue, New York, NY 10018 USA; 4 Labyrinth Global Health, Inc., 546 15th Ave NE, St Petersburg, FL 33704 USA; 5 Wildlife Conservation Society, Health Program, Bronx, NY USA; 6 Wildlife Conservation Society (WCS), Peru Program, Lima, Peru; 7 Global Health Program, Smithsonian’s National Zoological Park and Conservation Biology Institute, Washington, DC USA; 8 Mosaic/Global Viral Cameroon, Yaoundé, Cameroon; 9 Metabiota Inc, Nanaimo, VC Canada; 10 Institut Pasteur du Cambodge, 5 Monivong Blvd, PO Box 983, Phnom Penh, 12201 Cambodia; 11 Wuhan Institute of Virology, Chinese Academy of Sciences, Wuhan, China; 12 Kinshasa School of Public Health, University of Kinshasa, Kinshasa, Democratic Republic of the Congo; 13 Metabiota Inc., Kinshasa, Democratic Republic of the Congo; 14 Egypt National Research Centre, 12311 Dokki, Giza Egypt; 15 Aklilu Lemma Institute of Pathobiology, Addis Ababa University, Addis Ababa, Ethiopia; 16 Metabiota Cameroon Ltd, Yaoundé, Centre Region Avenue Mvog-Fouda Ada, Av 1.085, Carrefour Intendance, Yaoundé, BP 15939 Cameroon; 17 Military Veterinarian (Rtd.), P.O. Box CT2585, Accra, Ghana; 18 Centre de Recherche en Virologie (VRV) Projet Fievres Hemoraquiques en Guinée, BP 5680 Nongo/Contéya-Commune de Ratoma, Guinea; 19 Primate Research Center, Bogor Agricultural University, Bogor, 16151 Indonesia; 20 Faculty of Veterinary Medicine, Bogor Agricultural University, Darmaga Campus, Bogor, 16680 Indonesia; 21 Department Environment and Health, Institut Pasteur de Côte d’Ivoire, PO BOX 490, Abidjan 01, Ivory Coast; 22 Department of Basic Medical Veterinary Sciences, College of Veterinary Medicine, Jordan University of Science and Technology, Ar-Ramtha, Jordan; 23 Molecular Biology Laboratory, Institute of Primate Research, Nairobi, Kenya; 24 Department of Biochemistry, University of Nairobi, Nairobi, Kenya; 25 Conservation Medicine, Sungai Buloh, Selangor Malaysia; 26 Wildlife Conservation Society (WCS), Mongolia Program, Ulaanbaatar, Mongolia; 27 Center for Molecular Dynamics Nepal (CMDN), Thapathali -11, Kathmandu, Nepal; 28 Regional Headquarters, Mountain Gorilla Veterinary Project, Musanze, Rwanda; 29 Université Cheikh Anta Diop, BP 5005 Dakar, Sénégal; 30 Metabiota, Inc. Sierra Leone, Freetown, Sierra Leone; 31 Department of Veterinary Medicine and Public Health, College of Veterinary Medicine and Biomedical Sciences, Sokoine University of Agriculture, Morogoro, Tanzania; 32 Thai Red Cross Emerging Infectious Diseases Clinical Center, King Chulalongkorn Memorial Hospital, Bangkok, Thailand; 33 Wildlife Conservation Society (WCS), Bolivia Program, La Paz, Bolivia; 34 Facultad de Medicina Veterinaria y Zootecnia, Universidad Nacional Autónoma de México, México City, 04510 Mexico; 35 Centro de Biodiversidad y Genética, Universidad Mayor de San Simón, Cochabamba, Bolivia; 36 Laboratório de Epidemiologia e Geoprocessamento (EpiGeo), Instituto de Medicina Veterinária (IMV) Universidade Federal do Pará (UFPA), BR-316 Km 31, Castanhal, Pará 69746-360 Brazil; 37 Department of Microbiology, Sanjay Gandhi Post Graduate Institute of Medical Sciences, Lucknow, Uttar Pradesh India; 38 Wildlife Conservation Society (WCS), Vietnam Program, Hanoi, Vietnam; 39 Melbourne Veterinary School, Faculty of Veterinary and Agricultural Sciences, University of Melbourne, Werribee, VIC 3030 Australia; 40 Nyati Health Consulting, 2175 Dodds Road, Nanaimo, V9X0A4 Canada

**Keywords:** Ecological networks, Viral infection

## Abstract

Host-virus associations have co-evolved under ecological and evolutionary selection pressures that shape cross-species transmission and spillover to humans. Observed virus-host associations provide relevant context for newly discovered wildlife viruses to assess knowledge gaps in host-range and estimate pathways for potential human infection. Using models to predict virus-host networks, we predicted the likelihood of humans as hosts for 513 newly discovered viruses detected by large-scale wildlife surveillance at high-risk animal-human interfaces in Africa, Asia, and Latin America. Predictions indicated that novel coronaviruses are likely to infect a greater number of host species than viruses from other families. Our models further characterize novel viruses through prioritization scores and directly inform surveillance targets to identify host ranges for newly discovered viruses.

## Abstract (web summary)

Potential host range and spillover risk for novel viruses can be predicted using a network informed by known virus-host associations.

## Introduction

Identifying zoonotic virus emergence events at the earliest possible stage is key to mitigating outbreaks and preventing future epidemic and pandemic threats. By the time novel viruses are recognized in humans, often as a cluster of unusual cases, public health interventions to prevent or contain an epidemic face major challenges. However, determining the potential zoonotic transmission for newly discovered animal viruses, in the absence of documented human infection, is currently a major scientific challenge. New approaches are needed to evaluate and characterize the risk of zoonotic transmission of newly discovered animal viruses in the face of very limited data. Here we analyze human, domesticated animal, and wildlife surveillance and viral discovery data collected from 2009 to 2019, as part of a consortium-led One Health project aimed at strengthening pandemic threat detection capabilities in Africa, Asia, and Latin America^1^. Surveillance efforts resulted in 944 novel monophyletic clusters of virus sequences in wildlife (referred to as novel viruses henceforth) from 18 virus families sampled at high-risk animal-human disease transmission interfaces in 34 countries. As none of these viruses have yet been identified in humans, other indices were previously established to assess potential risk, such as virus host range or plasticity, and expert opinion based on integration of ecological and molecular characteristics of viruses^2–5^. We were able to quantify the risk of zoonotic transmission for 531 out of 944 novel animal viruses using data driven models to predict host-virus networks.

Patterns observed across host-virus networks have been used to understand virus sharing among vertebrate species^2,6,7^, and predict cryptic links between mammalian, and avian hosts and their viruses^8–10^. Host-virus network linkages can be informed by virus traits, virus biogeography, host ecological niches, and propensity for host sharing among viruses^10,11^. Precedence in viral sharing among species and ecological opportunities for spillover, as characterized by network topology, can inform propensities for newly discovered viruses that lack data^5^. Further exploration of these networks can aid in estimating the host plasticity of viruses, an important characteristic associated with zoonotic potential^2,5^. Unfortunately, systematically collected surveillance data to parameterize and validate these models have been missing^3^. Here, we apply a network approach to gain ecological insights from viruses that have been shared among species in nature and inform potential virus-host associations and zoonotic risk of novel viruses recently discovered from wildlife.

**Fig. 1:**
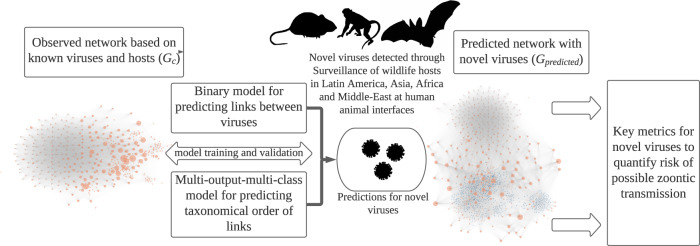
Modeling workflow. The figure shows the modeling procedure and methods implemented in the study. Orange dots represent a known virus in the observed (${G}_{c}$) and predicted networks (${G}_{{predicted}}$), blue dots represent novel viruses in the predicted network (${G}_{{predicted}}$). Virus-host networks: ${G}_{c}$, represents a unipartite observed network of known zoonotic and non-zoonotic viruses with nodes representing viruses and edges representing shared hosts. ${G}_{{predicted}}$represents the predicted unipartite network generated after predicting possible linkages between 531 novel viruses (blue) and known viruses. The node size is proportional to the betweenness centrality.

Using data from the literature, we developed a network that included 269 known zoonotic and 307 non-zoonotic viruses infecting 885 avian and mammalian hosts (${G}_{c}$; Fig. 1, Fig. S1). The network was used to train and validate two gradient boosting decision tree models to predict links and taxonomic orders of missing links generated by host sharing^12^. Trained models were used to predict possible host links for 531 novel viruses due to commonalities in host sharing with known viruses and generated a predicted host-virus network (${G}_{{predicted}}$, Fig. 1) formed due to the inclusion of novel viruses and their predicted linkages. We also predicted the taxonomic order of probable hosts shared as a link between two virus nodes of the network and the likelihood of the link to be humans, indicative of viruses’ predicted potential to be zoonotic.

## Results and discussion

### Virus-host network for known viruses (${{{{{{\boldsymbol{G}}}}}}}_{{{{{{\boldsymbol{c}}}}}}}$)

**Fig. 2:**
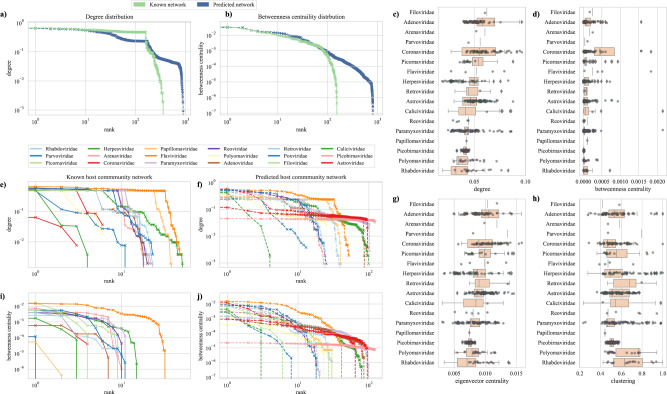
Predicting missing links between virus-host communities. Distribution shapes of degree (**a**) and betweenness centrality (**b**) for the observed and predicted network. Degree distributions for virus families in observed and predicted networks are shown in **e** and **f**. Similarly, shapes of betweenness centrality for virus families in observed and predicted networks are shown in **i** and **j**. Right panels show boxplots for novel virus families describing degree (**c**), betweenness centrality (**d**), eigenvector centrality (**g**), and clustering based on the predicted network formed by the binary prediction model (**h**).

We developed a unipartite network with viruses as nodes and host species as edges for all species recognized as a host for viruses based on data presented in previous studies and databases, specifically, data shared by Olival et al.,^4^ Pandit et al.,^3^ and Johnson et al.^13^ and GenBank. In the observed network (${G}_{c}$), viruses were represented as nodes and a link (edge) was generated if two viruses had been detected in the same host species. The observed network (${G}_{c}$) included 576 viruses as nodes and 35,838 edges (viruses linked because of shared hosts) representing 352 vertebrate species (Fig. 1). Exploration of network characteristics of known viruses revealed differences in host sharing among virus families. The distributions of centrality measures (Fig. 2a, b, e, i) for *Filoviridae*, *Flaviviridae*, *Hantaviridae*, and *Orthomyxoviridae* families were statistically different from the mean (Kolmogorov-Smirnov, *p* < 0.05). Furthermore, after accounting for sampling bias for individual viruses using PubMed hits (number of search results associated with virus using a specific search term), we ran a linear regression model with node-level permutations (10,000 permutations to further characterize the distribution of viruses within virus families in the network). Viruses in families *Hantaviridae*, *Filoviridae*, *Flaviviridae*, and *Orthomyxoviridae* had a significantly higher degree (*p* < 0.05) and eigenvector centrality (*p* < 0.05), indicating more connections in the host-virus network than other represented virus families. Viruses from the *Flaviviridae* family also had higher betweenness centrality (*p* = 0.01) indicating more connections based on shared host species (Figs. S2–S5). Results based on distributions of centrality measures, as well as node level regression models, show similar directionality for *Hantaviridae*, *Filoviridae*, *Flaviviridae*, and *Orthomyxoviridae* families across multiple network topological metrics. Our findings provide further evidence for direct relationship between higher host plasticity and greater zoonotic potential^2,4^.

The wildlife surveillance data consisted of tests for 99,375 animals, representing specimens from 861 species, mostly bats, rodents, primates, and other mammals (https://zenodo.org/record/5899054)^1^. To predict associations (linkages) between novel viruses with other viruses formed due to common host species, gradient boosting models were trained using network topological characteristics and families of viruses in the virus pairs to estimate: (1) whether virus pairs have a species host in common; and (2) the taxonomical order of shared hosts (Fig. 1).

### Characteristics of predicted network (${G}_{{predicted}}$) and newly discovered viruses

The binary model performed well in predicting the presence of links formed due to sharing of hosts between two virus nodes in the network (mean positive predictive value = 0.99, sensitivity = 0.96, F-score 0.97, Fig. S6). The distribution of predicted probability for all links using the binary model showed clear bimodal distribution (Fig. S7a). The accuracy scores as a function of precision and recall indicated good model performance beyond 0.15 predicted probability for the binary model (Fig. S8). Hence, as a more conservative approach and to give more weight to the precision, we decided to use 0.7 as an optimum threshold for detecting a positive link between two nodes (viruses). The performance of the multilabel model varied for taxonomical orders, with a high to moderate performance for predicting taxonomical group and order of ‘humans’ and Cetartiodactyla (Figs. S7, S9). For 531 novel viruses, we identified 184,055 possible links to other viruses formed due to sharing of hosts (based on the optimum probability threshold of 0.7 identified for the binary model) generating the predicted network (${G}_{{predicted}}$, Fig. 1, Fig. S7a). For these predicted links, between two viruses, the multiclass model was able to estimate the potential taxonomic order of the shared species for 175,113 links. For the remaining links, the model was not able to confidently predict a specific taxonomic order. Jaccard coefficient consistently showed high importance with all three importance metrics tested (gain, cover, and weight), indicating predictive ability in identifying missing links between unipartite viruses formed due to sharing of hosts (Fig. S10). Although, we suspect that these neighborhood-based topological features will be more predictive in a bipartite network setting due to more information flow between nodes.

Empirical biological networks are rarely scale-free (network with large hubs and showing a power-law distribution for degree)^14^ but a recently published study with host-host projected networks where links are represented by sharing of pathogens between hosts, has shown scale-free nature where models with power-law distributions showed the best fit for host-parasite networks^15^. Similarly, both observed (${G}_{c}$) and predicted (${G}_{{pr}{edicted}}$) networks provided evidence that some viruses shared significantly larger numbers of hosts, creating hubs of preferential attachment and showed weak evidence of scale-free nature (loglikelihood ratio test *p* > 0.05). The degree (Fig. 2a, e, f) and betweenness centrality (Fig. 2b, i, j) distributions for predicted network (${G}_{{predicted}}$) showed longer tails (Kolmogorov-Smirnov, *p* < 0.05) than the observed network (${G}_{c}$) both at network and virus family level. Mean network degree for all virus families reduced significantly with the addition of newly discovered viruses that were predicted to have fewer links than known viruses, indicating lower host plasticity for novel viruses than known viruses or insufficient adjustment of reporting bias (Fig. S11).

Based on a linear regression model with node-level permutations (10,000 permutations), our adjustment for search effort (PubMed hits) was found to have no effect on the degree (*p* = 0.39, Fig. S12) and betweenness centrality (*p* = 0.22, Fig. S13), but did significantly affect the eigenvector (*p* < 0.05, Fig. S14) and clustering coefficient (*p* < 0.05, Fig. S15) of novel viruses. These results indicate that sampling and reporting efforts affect our understanding of the predilection towards certain species as illustrated by clustering in the network, but do not affect the prediction of missing host links quantified by degree centrality within the network. Many of the newly discovered viruses were mostly detected in only one species (mean = 1.32, SD ± 0.99, *n* = 944). Long tails of centrality distributions generated for the predicted network (${G}_{{predicted}}$) and comparatively lower centrality measures for novel viruses, when compared with known viruses, support a tendency for newly discovered viruses to be more host-specific than previously recognized viruses, a pattern that should be further evaluated with additional sampling effort to identify the full host range for novel viruses.

Importantly, a comparison between virus families of novel viruses showed that novel coronaviruses had a higher degree (*p* < 0.001, Fig. 2c, Fig. S12), betweenness (*p* = 0.02, Fig. 2d, Fig. S13), and eigenvector (*p* < 0.001, Fig. S14) centralities in the predicted network compared to newly discovered viruses in all other virus families (Fig. 2c, d, g). In addition, the raw detection data showed significantly higher host diversity for novel coronaviruses with a mean of 2.02 (SD ± 2.03, *n* = 114) unique host species (maximum of 15 species) compared to 1.22 (SD ± 0.70, *n* = 834) for other novel viruses detected in this study. This finding raises concern about the ability of novel coronaviruses to infect a greater number of species than viruses from other families. The recently emerged SARS-CoV-2 and the previously emerged SARS-CoV-1, have shown a wide host breadth^16^. These predictions for novel coronaviruses highlight their key ecological properties that can influence spillover into humans. Following coronaviruses, novel flaviviruses showed significantly higher betweenness centrality (*p* < 0.001). Host taxonomic order for novel viruses had no significant association with the degree centrality of the virus in the predicted network. Predicted network characteristics not only differentiate virus families based on network characteristics but also predict network characteristics that are key in understanding the ecology of a novel virus and its behavior within the network community of hosts, including the expected breadth of host species most likely to be infected by that novel virus.

### Prioritizing novel viruses for further characterization

For the 531 novel viruses, we developed prioritization metrics that inform on the ecological and evolutionary tendencies for spillover based on number of human links with known viruses predicted by the multiclass model. Novel viruses from *Herpesviridae, Rhabdoviridae*, *Coronaviridae*, *Adenoviridae*, *Astroviridae*, and *Paramyxoviridae* families not only showed a high median probability of sharing human links with known viruses (Fig. S16) but also were predicted to have large numbers of human links in the predicted network (${G}_{{predicted}}$). Novel viruses of the *Picobirnaviridae* and *Rhabdoviridae* families detected here have been speculated to be hyper-parasites infecting bacteria and insects and were identified in mammalian host samples. Hence the predicted associations for these virus families should not be inferred as infection but only as detection in host samples (e.g., potentially insect viruses detected in oral swab samples from bats). Based on generalized linear mixed models, search effort (PubMed hits) was not associated with the predicted number of human links (*p* = 0.24, Table S1) nor the mean probability of sharing human links for novel viruses (*p* = 0.778, Table S2).

**Fig. 3:**
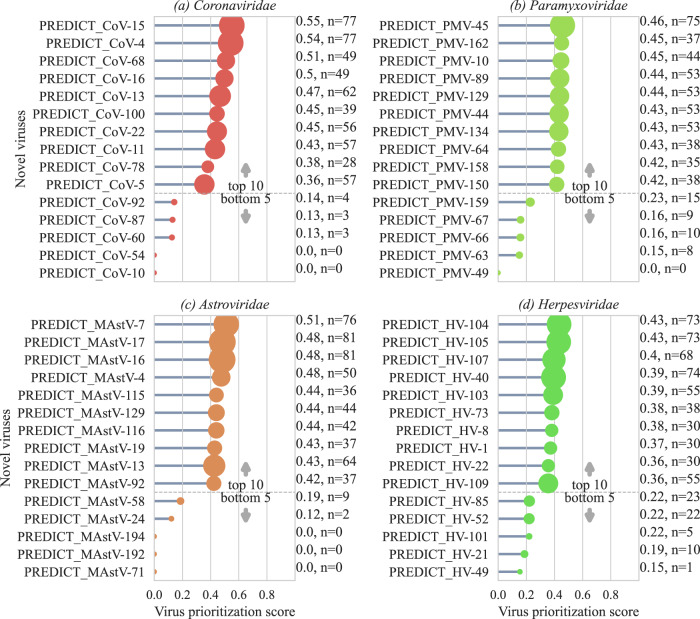
Prioritization metrics for novel viruses to understand zoonotic risk. Top ten and bottom five newly discovered viruses from six virus families (**a**–**d**) with the virus prioritization scores based on multiclass model predictions. Annotations show the score and support represented by the number of human links predicted.

For a relative comparison of zoonotic risk for novel viruses, a prioritization score was developed based on the predicted probability of links being human and the number of shared human links in the predicted network for a given virus. To understand the performance of the prioritization score, we compared scores for known zoonotic and non-zoonotic viruses generated by the ensemble of both binary and multi-class models. Results indicated significantly higher prioritization scores for known zoonotic viruses (Fig. S17, *p* < 0.001) compared to known non-zoonotic viruses. Prioritization scores were derived essentially from the prediction of new/yet unobserved network links generated by the virus with another virus formed due to sharing of hosts. However, models were unable to predict new links for well-recognized viruses that have numerous hosts, such as Rabies virus and West Nile virus, and consequently resulted in a prioritization score of zero. Figure 3a–d shows the top ten and bottom five novel viruses from four virus families for relative comparison based on the prioritization score (Figs. S18–S24). PREDICT_CoV-15 found in two *Phyllostomidae* bats from South America (*Artibeus lituratus*, *Sturnira lilium*) scored the highest prioritization score in all novel viruses. Other top ten novel coronaviruses based on the prioritization score included viruses detected in Phyllostomidae bats (PREDICT_CoV-4, PREDICT_CoV-13, PREDICT_CoV-11, PREDICT_CoV-5). Out of these, PREDICT_CoV-11 was also detected in Mormoopidae species (*Pteronotus personatus*) and PREDICT_CoV-5 was found in Vespertilionidae species (*Bauerus dubiaquercus*) during the surveillance. These also included coronaviruses detected in Southeast Asian Pteropodidae bat species such as PREDICT_CoV-16 and PREDICT_CoV-22. PREDICT_CoV-22 was also detected in Hipposideridae bat species (*Hipposideros lekaguli*). PREDICT_CoV-78 detected in multiple bat and rodent species of Southeast Asia also showed a high prioritization score. These model outcomes, especially the prioritization score, provide a data-driven tool to quantify zoonotic risk for novel viruses. Even though the model is trained on numerous data points for known zoonotic and non-zoonotic viruses, individual predictions for newly discovered viruses would only require data on hosts and virus family if used within our modeling framework.

### Prioritizing future surveillance

**Fig. 4:**
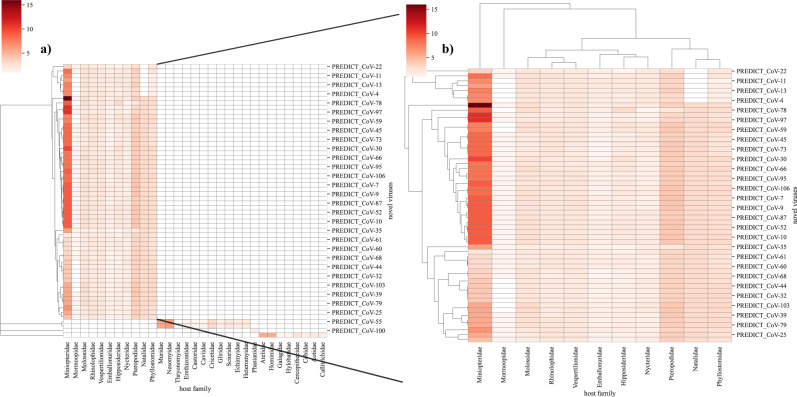
Surveillance targets for novel coronaviruses based on predicted sharing of hosts with known viruses. The red color represents the evidence of species in the taxonomic family (cumulative probability) with darker red color indicating a higher number of species occurrences from taxonomical families adjusted by model predicted probability. **a** shows clustering of novel coronaviruses by the host, and **b** focuses on novel coronaviruses found in bats. Clustering is based on the Bray-Curtis dissimilarity index.

The sharing of viruses among hosts is driven by geographical overlap and synergies in ecological niches of hosts, as well as virus-specific characteristics that enable cross-species transmission^10^. Novel viruses discovered in rodents, bats, primates, and other mammalian hosts were sampled from sites in close association with people, or at high-risk interfaces that can facilitate disease transmission in urban and rural settings^1,13^. Additional surveillance across a broader taxonomic range is essential to gain insights on newly detected viruses, further inform spillover risk, and improve model predictions presented here. We used our network model and host taxonomic data in which the novel virus is first detected to prioritize host species (surveillance targets) for further surveillance of newly discovered viruses (Supplementary Data 1). Moreover, given the recent SARS-CoV-2 pandemic we further explored surveillance targets for novel coronaviruses. Novel coronaviruses were detected in bats, rodents, birds, and primates (Fig. 4a). For novel coronaviruses, that were detected in bats, predicted surveillance targets for bat coronaviruses showed three distinct clusters (Fig. 4b). The first cluster of novel coronaviruses in bats had a higher proportion of predicted species from the *Miniopteridae* family (Bent-winged bats) but none from *Natalidae* (Neotropical funnel-eared bats). Another prominent cluster prioritized all 11 chiropteran families, while the third cluster of coronaviruses showed relatively fewer host recommendations from *Miniopteridae* bats. Representation of these surveillance targets through these clusters highlights host predilection of novel coronaviruses and indicates their preferential sharing of hosts. These clusters also support earlier results related to the scale-free nature of the predicted network (${G}_{{predicted}}$) by creating virus hubs in the virus-host network. Cluster maps for other virus families providing evidence for future surveillance are shown in Figs. S25–S31 and Supplementary Data 2.

Grange et al. developed a tool that ranks viruses for an animal to human spillover using a risk-based approach validated by inputs from various experts from the field of virology, epidemiology, and ecology^5^. Our approach, on the other hand, quantifies the risk of spillover agnostically and informs the predicted host range solely based on existing data available across the breadth of viruses and natural infections observed in free-ranging mammalian and avian hosts. Although numerous studies have been recently published that predict host-pathogen predilections, our framework quantifies the risk for viruses that have been recently discovered in animal hosts. Network models have shown to perform well with the inclusion of ecological trait data^10,17^ and genome sequences^18^, but, with the limited data available for novel viruses, the approach provided here is an important step towards characterizing zoonotic potential for newly discovered animal viruses in the face of sparse data. These results may imply that network models are better at identifying a predictive signal when they are virus-centric (viruses as nodes and shared hosts as edges), particularly given previous host-centric work has produced mixed results when using trait-agnostic network modelling approaches^17^. Our network approach presents some limitations specifically for viruses that have been detected in species with limited surveillance efforts to date and are thus not part of the training data. For this reason, we were able to generate predictions for only 531 novel viruses out of 944. The remaining 413 novel viruses without predictions were detected in species that were never found positive for any virus, starkly indicating the lack of surveillance in wildlife. Further, model findings should be interpreted as associations between hosts and viruses based on the detection of viruses in samples collected from host species. These associations require further understanding around the role of hosts in the transmission ecology of viruses, especially to elaborate if hosts can serve as reservoir, amplifying, or dead-end hosts. Detection of a virus in a host species is not always correlated with that host’s ability to produce viremia for further transmission. Similarly, some of the novel viruses from *Picobirnaviridae* and *Rhabdoviridae* have been speculated to be hyperparasites and the interpretation of these detections and predicted host-associations need further investigations.

## Conclusions

Novel viruses with high scores on the prioritization metrics present a strong eco-evolutionary case for further genetic and in-vivo characterization to understand the risk of spillover. The scoring will help streamline in-depth in-vivo characterization and develop additional hypotheses related to genetic and ecological mechanisms for cross-species transmission and zoonotic spillover. Nucleotide data associated with novel viruses presented here are short, hence the current model framework of using only host associations provides a key advantage. However, network models have shown to improve prediction capacities when nucleotide data are included as features for prediction^11^. These tools will improve with further surveillance and discovery of new viruses and their hosts^19^, ultimately informing our understanding of the mechanisms of zoonotic emergence for viruses from wildlife.

## Methods

### Data collection

Virus-host data was collated from various sources. Major sources for the association databases included data shared by Olival et al^4^., Pandit et al.^3^, and Johnson et al.^13^. In data provided by Olival et al (assessed September 2019), host-virus associations have been assigned a score, based on detection methods and tests that are specific and more reliable. We used associations that have been identified as the most reliable (stringent data) from Olival et al^4^. In addition, a query in GenBank was run to parse out hosts reported for each GenBank submission for viruses presented in each of these three databases. Initially, for each virus name, taxonomic ID was identified using e*ntrez.esearch* function in biopython package. The taxonomic ID helped linked to the GenBank databases, identify the ICTV lineage and associated data in PubMed^20,21^. NCBI TaxID closely follows the ICTV database, but some recent changes in ICTV might not always be reflected in NCBI, so we manually checked names to ensure matching. This included virus genus and family information along with a standard virus name. Host data were aggregated based on the taxonomic ID and associated standard name. Finally, for each virus, a search was completed in PubMed to compile the number of hits related to the virus and their vertebrate hosts using the search terms below. The number of PubMed hits (*PMH1*) were used as a proxy for sampling bias^3,13^. The virus-host association data source is presented in supplementary code and data files (https://zenodo.org/record/5899054).

$$	searchterm= (+virus\_name+\,[Title/Abstract])\\ 	AND\left(host\,OR\,hosts\,OR\,reservoir\,OR\,reservoirs\,OR \right.\\ 	wild\,OR\,wildlife\,OR\,domestic\,OR\,animal\,OR\,animals\,OR\\ 	mammal\,OR\,bird\,OR\,birds\,OR\,aves\,OR\,avian\,OR\,avians\\ 	\left. OR\,vertebrate\,OR\,vertebrates\,OR\,surveillance\,OR\,sylvatic\right)$$

Along with the PubMed terms we also queried the *nucleotide* database on PubMed using the taxonomic ID to find the number of GenBank entries for these viruses (*PMH2*). A correlation analysis between the *PMH1* and *PMH2* of well-recognized known viruses showed a high correlation with each other for us to safely use GenBank hits for novel viruses during the prediction stage of the model (Fig. S32).

### Development of ${{{{{{\boldsymbol{G}}}}}}}_{{{{{{\boldsymbol{c}}}}}}}$

a. Centrality measures of observed network (${{{{{{\boldsymbol{G}}}}}}}_{{{{{{\boldsymbol{c}}}}}}}$)

To test if centrality measures (degree centrality, betweenness centrality, eigenvector centrality, clustering coefficient) for viral nodes in the observed network (${G}_{c}$) vary significantly between viral families, we firstly used the Kolmogorov-Smirnov (KS) test. KS test is routinely used to identify distances between cumulative distribution functions of two probability distributions and is largely used to compare degree distributions of networks^22,23^. For each viral family, distributions of centrality measures (degree centrality, betweenness centrality, and eigenvector centrality) and clustering coefficient within the observed network (${G}_{c}$) were compared with the distribution of all nodes in the network using the two-tailed KS test. Secondly, a linear regression model with virus family as a categorical variable and the number of PubMed hits as a covariate to adjust for sampling bias were fitted to understand associations of viral families with centrality measures.

$${centrality}\,{measure}={\beta }_{0}{intercept}+{{\beta }_{1}{Viral}{family}}_{{categorical}}+{\beta }_{2}{PubMed}\,{hits}$$

After fitting the model, node-level permutations were implemented. For each random permutation, the output variable was randomly assigned to covariate values and the model was re-fitted. Finally, a *p-value* was calculated by comparing the distribution of coefficients from permutations with the original model coefficient.

### Network topology feature selection

Using the observed network (${{{{{{\boldsymbol{G}}}}}}}_{{{{{{\boldsymbol{c}}}}}}}$), multiple network topological features for all node (virus) pairs were calculated. The following are topological network features calculated. Features data type, definition and methods to calculate these features are presented in Table S3.

1. The Jaccard coefficient: a commonly used similarity metric between nodes in information retrieval, is also called an intersection of over the union for two nodes in the network. In the unipartite network generated here, it represents the proportion of common neighbor viruses from the union of neighbor viruses for two nodes. Neighbor viruses are defined as viruses with which the virus shares at least a single host.

2. Adamic/Adar (Frequency-Weighted Common Neighbors): Is the sum of inverse logarithmic degree centrality of the neighbors shared by two nodes in the network^24^. The concept of Adamic Adar index is a weighted common neighbors for viruses in the network. Within network prediction, the index assumes that viruses with large neighborhoods have a less significant impact while predicting a connection between two viruses compared with smaller neighborhoods.

Both Jaccard and Adamic Adar coefficients have been routinely used for generalized network prediction and have shown high accuracy in predicting missing links in networks, specifically bipartite networks^25^, the information flowing through neighborhoods formed by two nodes might not always be enough to have similar predictive power in an unipartite network. This warrants use of other topology features along with neighborhood-based features.

3. Resource allocation: Similarity score of two nodes defined by the weights of common neighbors of two nodes. Resource allocation is another measure to quantify the closeness of two nodes in the network and hence to understand the similarity of hosts they infect.

4. Preferential attachment coefficients: The mechanism of preferential attachment can be used to generate evolving scale-free networks, where the probability that a new link is connected to node *x* is proportional to *k*^26^.

5. Betweenness centrality: For a node in the network betweenness centrality is the sum of the fraction of all-pairs shortest paths that pass through it. The feature that we used for training the supervised learning model was the absolute difference between of betweenness centralities of two nodes. The difference between the betweenness centrality represents the difference in the sharing observed by two viruses in the pair.

6. Degree centrality: The degree centrality for a node *v* is the fraction of nodes it is connected to. The feature that we used for training the supervised learning model was the absolute difference between degree centralities of two nodes. Unlike the difference in the betweenness centrality, the difference in degree centrality only looks at the difference in the number of observed host sharing.

7. Network clustering: All nodes were classified into community clusters using Louvain methods^27^. A binary feature variable was generated to describe if both the nodes in the pair were part of the same cluster or not. If both viruses are from the same cluster, it represents a similar host predilection than when both viruses are not from the same cluster hence accounting for the evolutionary predilection of viruses (or virus families) to infect a certain type of host.

These topological network characteristics come with certain limitations when it comes to the unipartite network of viruses with links formed due to shared hosts and might not truly represent the flow of information between nodes as compared to a bipartite network. Therefore, to account for these limitations, we use multiple network features as weak learners in our model building characteristics summarizing the network through the use of several quantitative metrics. In addition to this, we estimated the feature importance of these metrics in predicting missing links between viruses to quantify the information pasting through these links.

Pearson’s correlation coefficients were calculated to identify highly correlated features and for choosing features for model training (Fig. S33). Virological features included in model training were categorical variables describing the virus family of both the nodes in the pair, followed by a binary variable if both the viruses belong to the same virus family. During the model development, PubMed hits generated three predictive features for each pair of viruses on which model training and predictions were conducted. These included two features representing PubMed hits for the two viruses in the pair (PubMed_V1_, PubMed_V2_) and the absolute difference between PubMed_V1_ and PubMed_V2_ to account for differences in sampling bias between the two viruses.

### Cross-validation and fitting generalized boosting machine (GBMs) models

A nested-cross-validation was implemented for the binary model while simple cross-validation was implemented for the multiclass model (multiple output categories). The parameters of the binary model were first hyper-tuned using a cross-validated grid-search method. Values were tested using a grid search to find the best-performing model parameters that showed the highest sensitivity (recall). The parameters tested for hypertuning and their performance are provided in the supplementary material (supplementary results and Table S5). For further cross-validation of the overall binary model, all the viruses were randomly assigned to five groups. For each fold, the viruses assigned to a group were dropped from the data, and a temporary training network (${{{{{{\boldsymbol{G}}}}}}}_{{{{{{\boldsymbol{t}}}}}}}{{{{{\boldsymbol{)}}}}}}$ was constructed, assuming that this represented the current observed status of the virus-host community. For all possible pairs in ${{{{{{\boldsymbol{G}}}}}}}_{{{{{{\boldsymbol{t}}}}}}}$ (both that sharing and not sharing any hosts) ten topological and viral characteristics were calculated as training features (Table S4). Categorical features were one-hot-encoded and numeric features were scaled. An XGBClassifier model with binary: logistic family was trained using the feature dataset to predict if virus pairs share hosts (1,0 encoded output). The cross-validation was also used to determine the optimum decision threshold for determining binary classification (Fig. S6) and a precision-recall curve was used to identify positive predictive value and sensitivity at the optimum threshold (Fig. S8).

The multiclass model was implemented in the same way, creating an observed network (${G}_{c}$) based on species-level sharing of hosts and randomly dropping viruses to generate a training network (${G}_{t}$) to train the XGboost model. The output variables were generated based on the taxonomical orders of shared hosts. A pair of viruses can share multiple hosts, hence we trained a multioutput-multiclass model. Humans were considered an independent category of taxonomical order (label) and were given a separate label from primates. For fine-tuning the multiclass model, we started with the best performing parameters of the binary model and manually tested 5 combinations of model parameters by adjusting values of the learning rate, number of estimators, maximum depth, and minimum child weight (Supplementary code and results).

We used three methods to estimate the importance of features for our binary model. Specifically, improvement in accuracy brought by branching based on the feature (gain), the percentage of times the feature appears in the XGboost tree model (weight), and the relative number of observations related to the specific feature (cover). Results for feature importance are shown in supplementary results (Fig. S10).

### Missing links for novel viruses, binary and multiclass prediction

The wildlife surveillance data represented a sampling of 99,379 animals (94,723 wildlife, 4656 domesticated animals) conducted in 34 countries around the world between 2009–2019 (Table S6)^1^. Specimens were tested using conventional Rt-PCR, Quantitative PCR, Sanger sequencing, and Next Generation Sequencing protocols to detect viruses from 28 virus families or taxonomic groups (Table S7). Testing resulted in 951 novel monophyletic clusters of virus sequences (referred to as novel viruses henceforth). Within 951 novel viruses, 944 novel viruses had vertebrate hosts that were identified with certainty based on barcoding methods and field identification. Host species identification was confirmed by cytochrome b (cytb) DNA barcoding using DNA extracted from the samples^28^. We predicted the shared host links between novel viruses and known viruses using binary and multiclass models in the following steps. Out of 944 novel viruses discovered in the last ten years, we were able to generate predictions for 531 novel viruses that were detected in species already classified as hosts within the network. The remaining 413 viruses were the first detection of any virus in that species and thus host associations could not be informed by the observed network (${{{{{{\boldsymbol{G}}}}}}}_{{{{{{\boldsymbol{C}}}}}}}$) data.

1. A new node representing the novel virus was inserted in the observed network (${{{{{{\boldsymbol{G}}}}}}}_{{{{{{\boldsymbol{c}}}}}}}$). Using the list of species in which the novel virus was detected, new edges were created with known viruses that are also known to be found in those hosts. This generated a temporary network for the novel virus (${{{{{{\boldsymbol{G}}}}}}}_{{temp}}$). If the novel virus was not able to generate any edges with known viruses, meaning the host in which they have been found was never found positive for any known virus, predictions were not performed.

2. Using ${{{{{{\boldsymbol{G}}}}}}}_{{temp}}$ feature values were calculated for the novel virus (betweenness centrality, clustering, and degree). For all possible pairs of the novel virus with known viruses that are not yet connected with each other through an edge in ${{{{{{\boldsymbol{G}}}}}}}_{{temp}}$ a feature dataset was generated (Jaccard coefficient_(novel virus, known virus)_, the difference in betweenness centrality of the novel virus and known virus, if the novel virus and known virus were in the same cluster, the difference in degree centrality_(novel virus, known virus)_, if the novel virus and known virus were from same virus family, the difference in PubMed hits_(novel virus, known virus)_, PubMed hits for the novel virus, PubMed hits for the known virus). Studies and nucleotide sequences for novel viruses are expected to be published and shared on PubMed’s Nucleotide database and in various peer-reviewed publications. Data associated with GenBank accession numbers and nucleotide sequences for novel viruses are presented in Supplementary Data 3 and Supplementary Data 4 respectively. At the time of development of the model, data for all viruses was not shared in a format that would reflect on PubMed’s database, we decided to use the number of unique species the virus was detected in the last ten years of wildlife surveillance conducted by the USAID PREDICT project. These detections will be reflected in PubMed’s Nucleotide database and search term eventually, hence we considered them as a proxy for search terms conducted for known viruses. Currently, evaluation of the effects of this substitution of PubMed hits with the number of detections for novel viruses is not possible with limited data on novel viruses but needs to be reevaluated as more studies are published on these novel viruses. To further evaluate the association between PubMed hits through search term and Genbank hits, we ran a generalized linear regression model with PubMed hits as dependent variable and Genbank hits as intendent variable, accounting for virus families.

$${{PubMed}}_{{Search}}\left({\log }\right)={\beta }_{0}{intercept}+{{\beta }_{1}{Virus}{family}}_{{categorical}}+{\beta }_{2}{Genbank}\,{hits}\,({\log })$$

The results indicated that Genbank hits had statistically significant predictive value in predicting PubMed hits (*β* = 0.72, *p* < 0.005) even after accounting for various virus families. Multiple virus families showed statistically different estimates than the reference virus family (*Adenoviridae*) indicating a significantly different association than other virus families. Results of the generalized linear regression model are presented in Table S8.

3. Using this dataset for the novel virus, a binary presence of a link between the novel virus and known viruses was predicted using the trained binary model. The taxonomic order of the host link was predicted using the trained multiclass model.

4. For each possible link, the binary model predicted the probability of sharing a link, and the multiclass model predicted multivariate outcomes of taxonomic orders and associated probabilities. A threshold of 0.70 for the binary prediction model was used to classify if the link is present or not and only those links were explored for their corresponding multiclass model outputs.

5. The multiclass model showed higher performance for correctly classifying links as “human” hosts than other numerous avian and mammalian taxonomic orders. Hence, the multiclass model outputs were summarized into either humans or other taxonomic groups. For the novel virus, a list of known viruses with the predicted link was generated. Using the hosts of these known viruses and the taxonomic order in which the novel virus was detected, a list of most likely species was generated based on the overall frequency of the host species. For understanding the likelihood of infecting humans two factors were considered to be of importance. Firstly, the number of links where humans are predicted as shared hosts with known viruses ($n$) and the average model-predicted probability of those links. A representation was generated incorporating the probability and available model support in terms of number links to reflect the likelihood and compare viruses relative to each other.

To test if virus family, the taxonomic order of hosts in which novel viruses were detected, and the number of times the viruses were detected (equivalent to PubMed hits for known viruses) influenced node (virus) level network centrality measures in the predicted network (${{{{{{\boldsymbol{G}}}}}}}_{{{{{{\boldsymbol{p}}}}}}}$) a linear regression model was fitted with centrality measures.

$${centrality}\,{measure}=	\ {\beta }_{0}{intercept}+{{\beta }_{1}{Viral}{family}}_{{categorical}}\\ 	+{{\beta }_{2}{Host}{Order}}_{{categorical}}+{\beta }_{3}{PubMed}\,{hits}$$

For each of the random 10,000 node-level permutations, the output variable (centrality measure) was randomly assigned to covariate values and the model was re-fitted. A *p-value* was calculated by comparing the distributions of coefficients with the original model coefficient. These models were fitted for degree centrality, betweenness centrality, eigenvector centrality, and clustering coefficient of novel viruses in the predicted network.

### Prioritization score for novel viruses

Generalized Linear Mixed Models were used to understand the association effects of virus family, taxonomic order of the host and PubMed hits on the number of predicted human links and mean probability of the predicted links. The models were fit using *glmmTMB* and *glm* packages in R. For relative comparison of zoonotic risk and for prioritizing novel viruses for further characterization, a prioritization metric was developed based on the predicted probability of sharing the humans as hosts with known viruses (${p}_{{sharing\; humans}}$) and the number of predicted shared human links (${n}_{{humans}}$) in the predicted network for the given virus (${G}_{{predicted}}$). Distributions for both ${p}_{{sharing\; humans}}$ and ${n}_{{humans}}$ were normalized and multiplied to generate a single score for a virus and for appropriate relative comparisons between novel viruses. To understand the behavior of the prioritization score when predicting the zoonotic risk of novel viruses, we also compared prioritization scores of known zoonotic and non-zoonotic viruses using the Kolmogorov-Smirnov test.

### Reporting summary

Further information on research design is available in the Nature Research Reporting Summary linked to this article.

## Supplementary information

Supplementary Materials

Description of Additional Supplementary Files

Supplementary Data 1

Supplementary Data 2

Supplementary Data 3

Supplementary Data 4

Reporting Summary

## Data Availability

Data reported in this paper are available at https://zenodo.org/record/5899054, https://data.usaid.gov/d/tqea-hwmr and https://data.usaid.gov/d/x3ij-fnrb, https://data.usaid.gov/Global-Health-Security-in-Development-GHSD-/PREDICT-Emerging-Pandemic-Threats-Project/tqea-hwmr.

## Code availability

Code used to develop models and generate results and figures presented in the paper is available at https://zenodo.org/record/5899054.
