# Author Correction: Predicting the potential for zoonotic transmission and host associations for novel viruses

**DOI:** 10.1038/s42003-022-04364-y

**Published:** 2023-01-10

**Authors:** Pranav S. Pandit, Simon J. Anthony, Tracey Goldstein, Kevin J. Olival, Megan M. Doyle, Nicole R. Gardner, Brian Bird, Woutrina Smith, David Wolking, Kirsten Gilardi, Corina Monagin, Terra Kelly, Marcela M. Uhart, Jonathan H. Epstein, Catherine Machalaba, Melinda K. Rostal, Patrick Dawson, Emily Hagan, Ava Sullivan, Hongying Li, Aleksei A. Chmura, Alice Latinne, Christian Lange, Tammie O’Rourke, Sarah Olson, Lucy Keatts, A. Patricia Mendoza, Alberto Perez, Cátia Dejuste de Paula, Dawn Zimmerman, Marc Valitutto, Matthew LeBreton, David McIver, Ariful Islam, Veasna Duong, Moctar Mouiche, Zhengli Shi, Prime Mulembakani, Charles Kumakamba, Mohamed Ali, Nigatu Kebede, Ubald Tamoufe, Samuel Bel-Nono, Alpha Camara, Joko Pamungkas, Kalpy J. Coulibaly, Ehab Abu-Basha, Joseph Kamau, Soubanh Silithammavong, James Desmond, Tom Hughes, Enkhtuvshin Shiilegdamba, Ohnmar Aung, Dibesh Karmacharya, Julius Nziza, Daouda Ndiaye, Aiah Gbakima, Zikankuba sajali, Supaporn Wacharapluesadee, Erika Alandia Robles, Benard Ssebide, Gerardo Suzán, Luis F. Aguirre, Monica R. Solorio, Tapan N. Dhole, Nguyen T. T. Nga, Peta L. Hitchens, Damien O. Joly, Karen Saylors, Amanda Fine, Suzan Murray, William B. Karesh, Peter Daszak, Jonna A. K. Mazet, PREDICT Consortium, Cátia Dejuste de Paula, Christine K. Johnson

**Affiliations:** 1 One Health Institute, School of Veterinary Medicine, University of California, Davis, Davis, CA 95616 USA; 2 Center for Infection and Immunity, Columbia University, New York, NY 10032 USA; 3 EcoHealth Alliance, 520 Eighth Avenue, New York, NY 10018 USA; 4 Labyrinth Global Health, Inc., 546 15th Ave NE, St Petersburg, FL 33704 USA; 5 Wildlife Conservation Society, Health Program, Bronx, NY USA; 6 Wildlife Conservation Society (WCS), Peru Program, Lima, Peru; 7 Global Health Program, Smithsonian’s National Zoological Park and Conservation Biology Institute, Washington, DC USA; 8 Mosaic/Global Viral Cameroon, Yaoundé, Cameroon; 9 Metabiota Inc, Nanaimo, VC Canada; 10 Institut Pasteur du Cambodge, 5 Monivong Blvd, PO Box 983, Phnom Penh, 12201 Cambodia; 11 Wuhan Institute of Virology, Chinese Academy of Sciences, Wuhan, China; 12 Kinshasa School of Public Health, University of Kinshasa, Kinshasa, Democratic Republic of the Congo; 13 Metabiota Inc., Kinshasa, Democratic Republic of the Congo; 14 Egypt National Research Centre, 12311 Dokki, Giza Egypt; 15 Aklilu Lemma Institute of Pathobiology, Addis Ababa University, Addis Ababa, Ethiopia; 16 Metabiota Cameroon Ltd, Yaoundé, Centre Region Avenue Mvog-Fouda Ada, Av 1.085, Carrefour Intendance, Yaoundé, BP 15939 Cameroon; 17 Military Veterinarian (Rtd.), P.O. Box CT2585, Accra, Ghana; 18 Centre de Recherche en Virologie (VRV) Projet Fievres Hemoraquiques en Guinée, BP 5680 Nongo/Contéya-Commune de Ratoma, Guinea; 19 Primate Research Center, Bogor Agricultural University, Bogor, 16151 Indonesia; 20 Faculty of Veterinary Medicine, Bogor Agricultural University, Darmaga Campus, Bogor, 16680 Indonesia; 21 Department Environment and Health, Institut Pasteur de Côte d’Ivoire, PO BOX 490, Abidjan 01, Ivory Coast; 22 Department of Basic Medical Veterinary Sciences, College of Veterinary Medicine, Jordan University of Science and Technology, Ar-Ramtha, Jordan; 23 Molecular Biology Laboratory, Institute of Primate Research, Nairobi, Kenya; 24 Department of Biochemistry, University of Nairobi, Nairobi, Kenya; 25 Conservation Medicine, Sungai Buloh, Selangor Malaysia; 26 Wildlife Conservation Society (WCS), Mongolia Program, Ulaanbaatar, Mongolia; 27 Center for Molecular Dynamics Nepal (CMDN), Thapathali -11, Kathmandu, Nepal; 28 Regional Headquarters, Mountain Gorilla Veterinary Project, Musanze, Rwanda; 29 Université Cheikh Anta Diop, BP 5005 Dakar, Sénégal; 30 Metabiota, Inc. Sierra Leone, Freetown, Sierra Leone; 31 Department of Veterinary Medicine and Public Health, College of Veterinary Medicine and Biomedical Sciences, Sokoine University of Agriculture, Morogoro, Tanzania; 32 Thai Red Cross Emerging Infectious Diseases Clinical Center, King Chulalongkorn Memorial Hospital, Bangkok, Thailand; 33 Wildlife Conservation Society (WCS), Bolivia Program, La Paz, Bolivia; 34 Facultad de Medicina Veterinaria y Zootecnia, Universidad Nacional Autónoma de México, México City, 04510 Mexico; 35 Centro de Biodiversidad y Genética, Universidad Mayor de San Simón, Cochabamba, Bolivia; 36 Laboratório de Epidemiologia e Geoprocessamento (EpiGeo), Instituto de Medicina Veterinária (IMV) Universidade Federal do Pará (UFPA), BR-316 Km 31, Castanhal, Pará 69746-360 Brazil; 37 Department of Microbiology, Sanjay Gandhi Post Graduate Institute of Medical Sciences, Lucknow, Uttar Pradesh India; 38 Wildlife Conservation Society (WCS), Vietnam Program, Hanoi, Vietnam; 39 Melbourne Veterinary School, Faculty of Veterinary and Agricultural Sciences, University of Melbourne, Werribee, VIC 3030 Australia; 40 Nyati Health Consulting, 2175 Dodds Road, Nanaimo, BC V9X0A4 Canada

**Keywords:** Ecological networks, Viral infection

Correction to: *Communications Biology* 10.1038/s42003-022-03797-9, published online 19 August 2022

In this Article, the first names of the authors were accidentally provided only as initials. The full names have now been added.

