# Supplementary material for: Predicting the potential for zoonotic transmission and host associations for novel viruses
Source: Commun Biol. 2022 Aug 19;5:844. doi: 10.1038/s42003-022-03797-9 (PMC9390964; doi:10.1038/s42003-022-03797-9)
Supplement: Supplementary file 1 — Supplementary Materials [file 42003_2022_3797_MOESM1_ESM.pdf]

Supplementary Materials for

Predicting the potential for zoonotic transmission and host associations for novel viruses

P. S. Pandit[1*], S.J. Anthony[2**], T. Goldstein[1**], K. J. Olival[3], M. M. Doyle[1], N. R. Gardner[1], B. Bird[1], W. Smith[1], D. Wolking[1], K. Gilardi[1], C. Monagin[1], T. Kelly[1], M. Uhart[1], J. H. Epstein[3], C. Machalaba[3], M. K. Rostal[3], P. Dawson[3], E. Hagan[3], A. Sullivan[3], H. Li[3], A. A. Chmura[3], A. Latinne[3], C. Lange[4], T. O'Rourke[4], S. Olson[5], L. Keatts[1], P. Mendoza[5], A. Perez[5], C. Dejuste de Paula[5], D. Zimmerman[6], M. Valitutto[6], M. LeBreton[7], D. McIver[8], A. Islam[3], V. Duong[9], M. Mouiche[7], Z. Shi[10], P. Mulembakani[11], C. Kumakamba[12], M. Ali[13], N. Kebede[14], U. Tamoufe[15], S. Bel-Nono[16], A. Camara[17], J. Pamungkas[18, 19], K. Coulibaly[20], E. Abu-Basha[21], J. Kamau[22], S. Silithammavong[8], J. Desmond[3], T. Hughes[3,23], E. Shiilegdamba[24], O. Aung[6], D. Karmacharya[25], J. Nziza[26], D. Ndiaye[27], A. Gbakima[28], Z. Sijali[29], S. Wacharapluesadee[30], E. Alandia Robles[31], B. Ssebide[26], G. Suzán[32], L. F. Aguirre[33], M. R. Solorio[34], T. N. Dhole[35], N. T. T. Nga[36], P. L. Hitchens[37], D. O. Joly[38], K. Saylors[4], A. Fine[5], S. Murray[7], W. Karesh[3], P. Daszak[3], J. A. K. Mazet[1], PREDICT Consortium, & C. K. Johnson[1*]

*Corresponding author: P.S. Pandit, pspandit@ucdavis.edu and C. K. Johnson, ckjohnson@ucdavis.edu

Supplementary Results

Feature selection based on Pearson's correlation coefficient:
Resource allocation index, Jaccard coefficient, Adamic Adar, and preferential attachment coefficient were highly correlated with each other (R^2>0.95), hence we decided to use only the Jaccard coefficient as a feature in training the model (Fig. S7).

Hypertuning binary model:
Grid search for tuning the best model parameters for the binary model resulted in the following parameters that performed the best for the XGBoost classifier.

```
XGBClassifier(base_score=0.5, booster='gbtree', colsample_bylevel=1,
       colsample_bytree=0.7, gamma=0.1, learning_rate=0.2,
       max_delta_step=0, max_depth=12, min_child_weight=3, missing=None,
       n_estimators=100, n_jobs=1, nthread=None,
       objective='binary:logistic', random_state=0, reg_alpha=0,
       reg_lambda=1, scale_pos_weight=1, seed=None, silent=True,
       subsample=1)
```

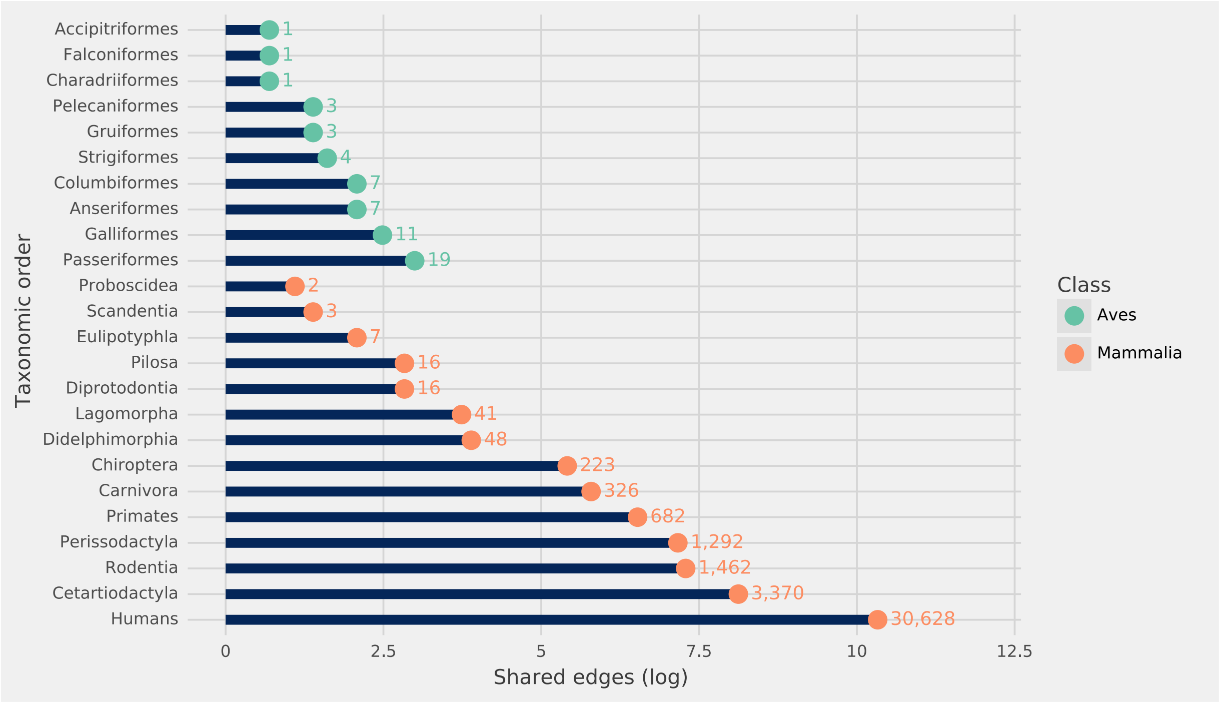

Fig. S 1: Distribution of edges by avian and mammalian taxonomic orders in the unipartite complete network ($\mathbf{G_c}$) generated by sharing of hosts by zoonotic and non-zoonotic viruses.

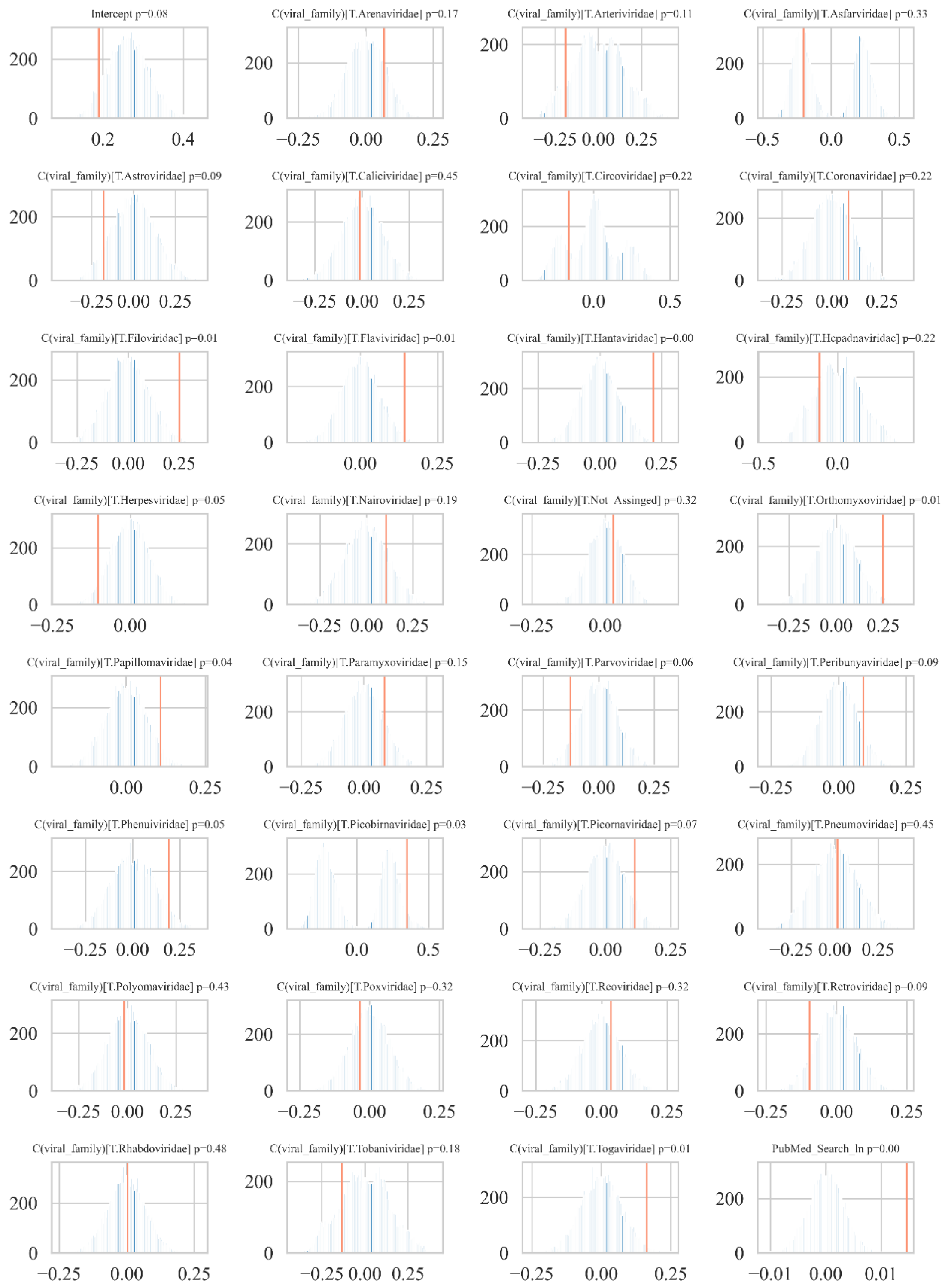

Fig. S2. Results of permutation-based regression analysis to understand the effects of virus family and citations on the degree centrality of viruses in the observed network. Blue lines show the distribution of coefficients after 10,000 random permutations. Red lines show original coefficients.

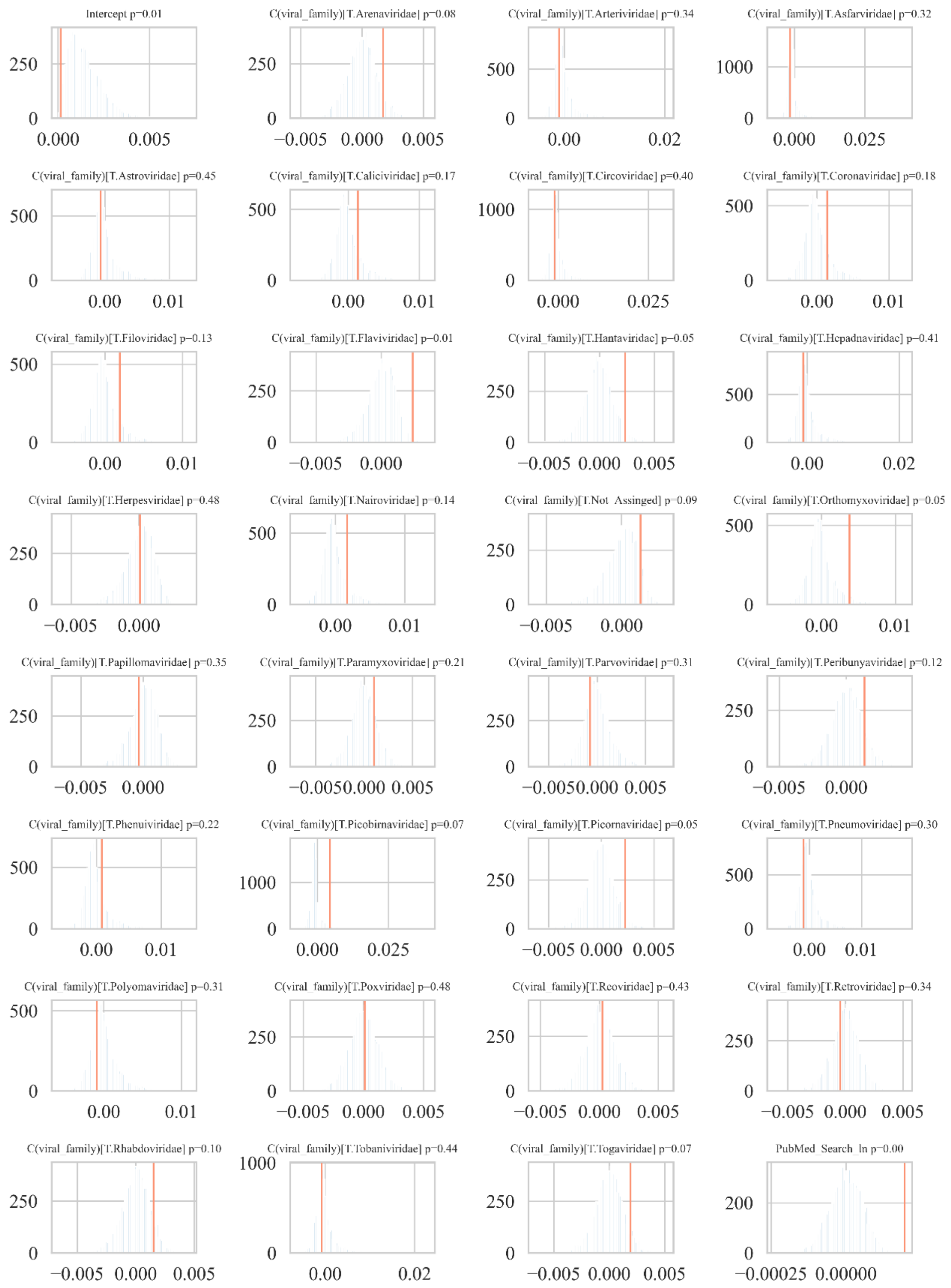

Fig. S3. Results of permutation-based regression analysis to understand the effects of viral family and citations on the betweenness centrality of viruses in the observed network. Blue lines show the distribution of coefficients after 10,000 random permutations. Red lines show original coefficients.

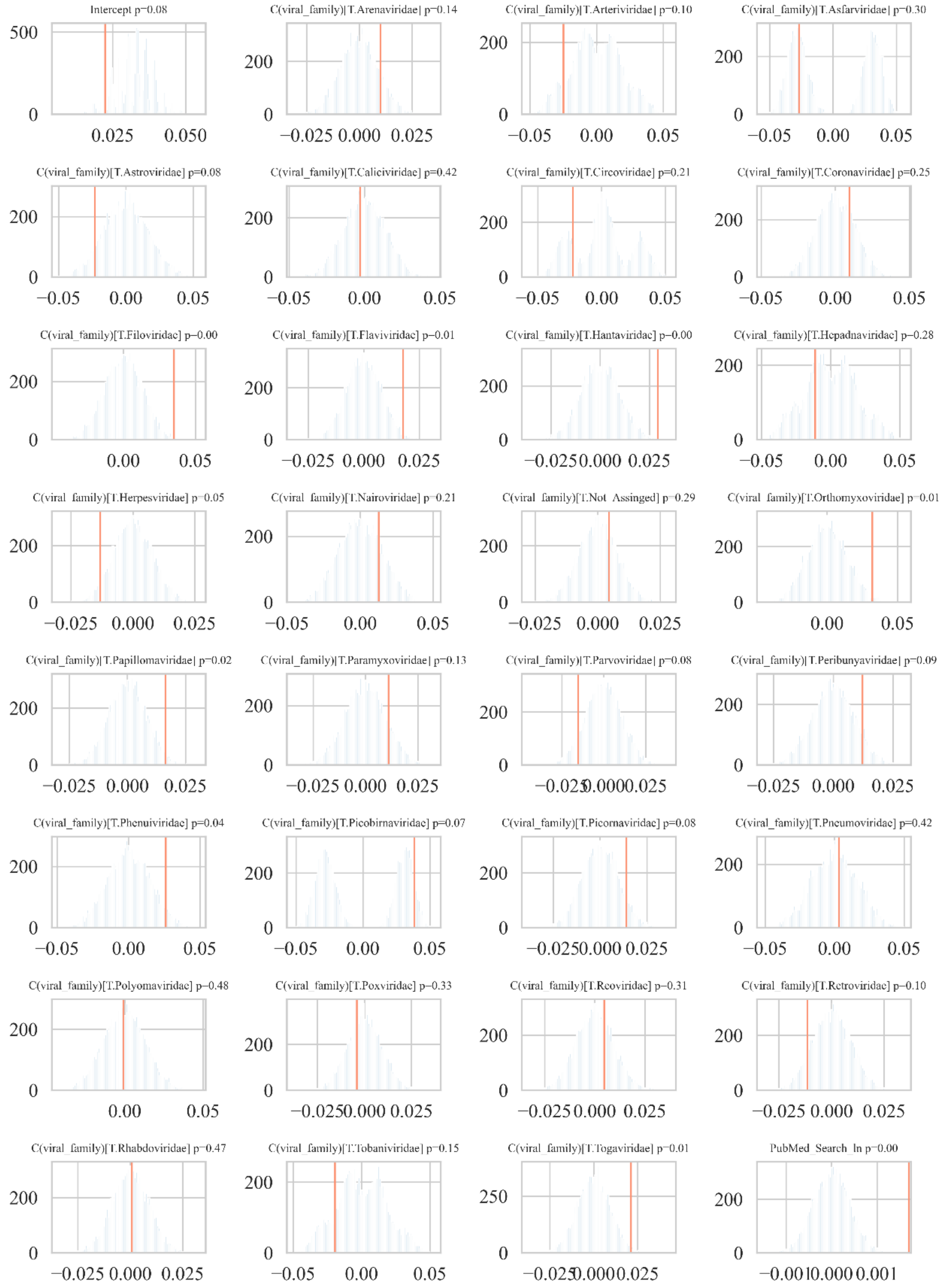

Fig. S4. Results of permutation-based regression analysis to understand the effects of viral family and citations on the eigenvector centrality of viruses in the observed network. Blue lines show the distribution of coefficients after 10,000 random permutations. Red lines show original coefficients.

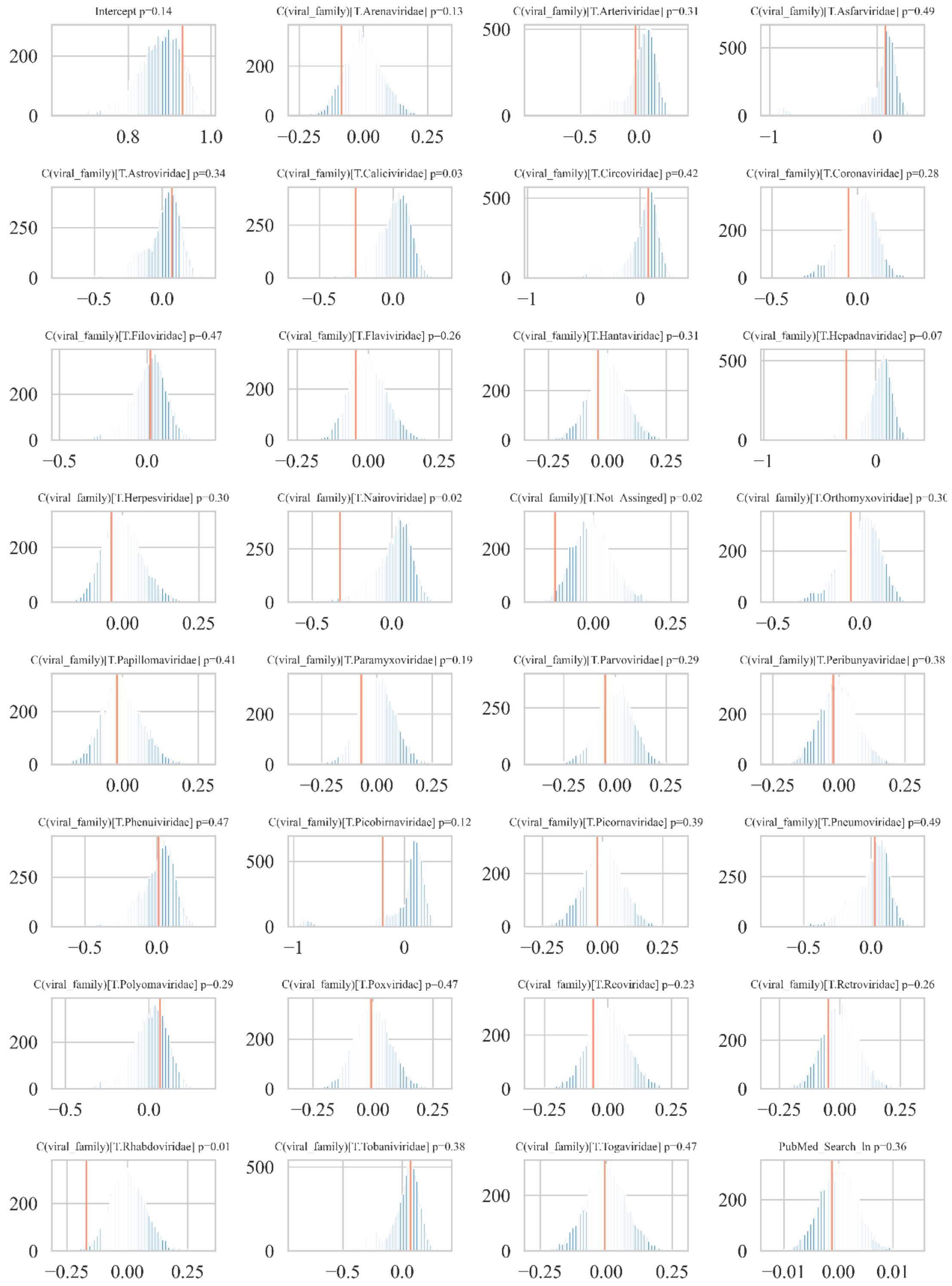

Fig. S 5. Results of permutation-based regression analysis to understand the effects of viral family and citations on the clustering coefficient of viruses in the observed network. Blue lines show the distribution of coefficients after 10,000 random permutations. Red lines show original coefficients.

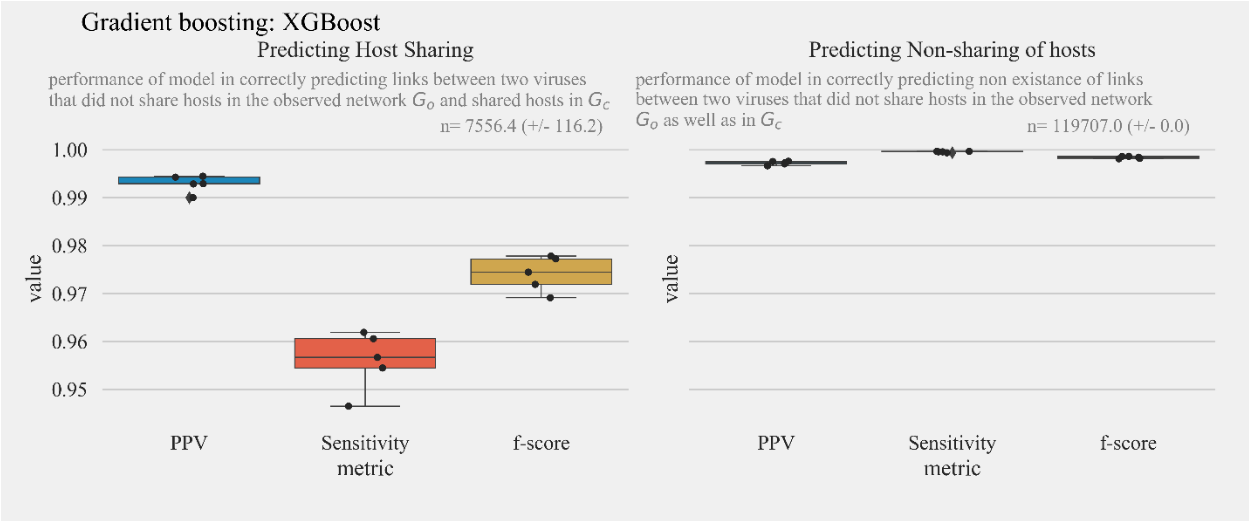

Fig. S 6. Sensitivity, positive predictive value, and f-score for correctly predicting the sharing of hosts in $\boldsymbol{G_c}$ by viral pairs that did not share hosts in $\boldsymbol{G_o}$ and non-sharing of hosts in $\boldsymbol{G_c}$ by virus pairs that did not share hosts in $\boldsymbol{G_o}$ for binary XGBoost model.

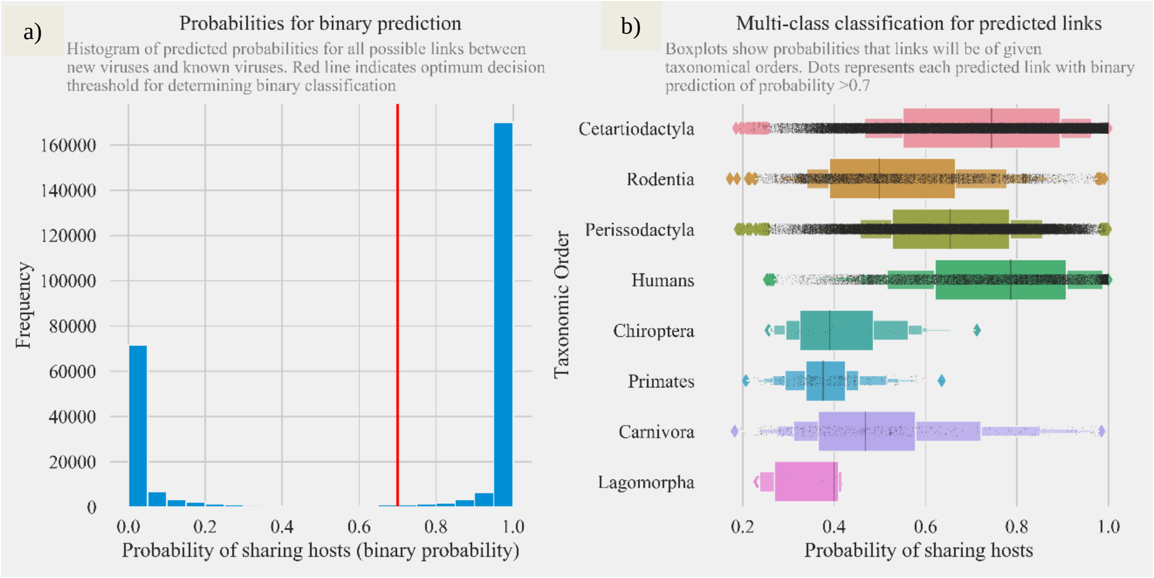

Fig. S 7: Predictions for PREDICT viruses. a) Histogram of predicted probabilities for the presence of a link between all possible pairs of PREDICT viruses and known zoonotic and non-zoonotic viruses (left panel). The red line indicates an optimum decision threshold for determining binary classification. On the right b), boxplots show predicted probabilities that the links predicted by the binary model between viruses are generated by the sharing of hosts from taxonomic order.

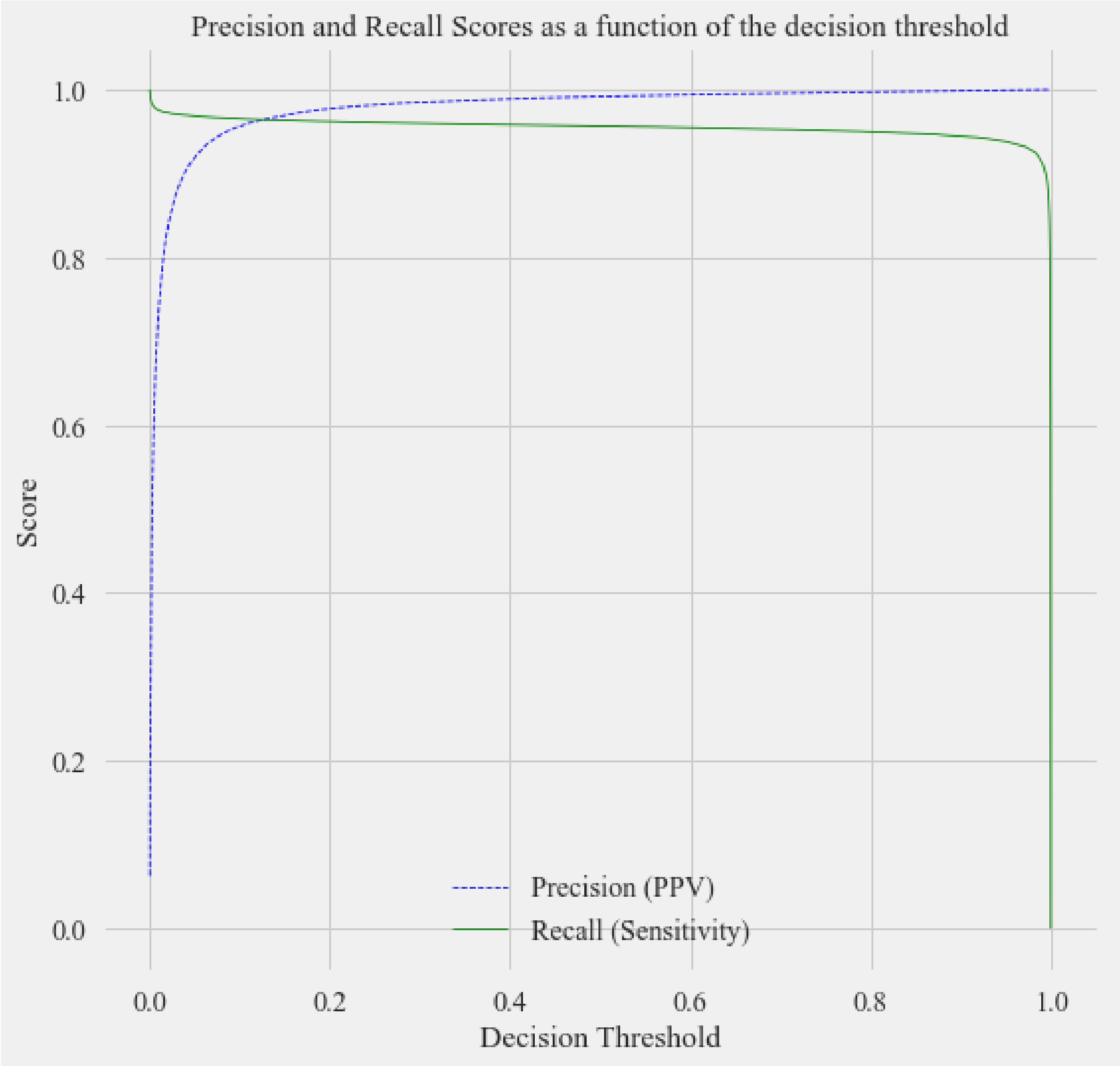

Fig. S 8: Precision recall scores as a function of the decision threshold shows a high score (beyond 0.15) model predicted probability for the binary model.

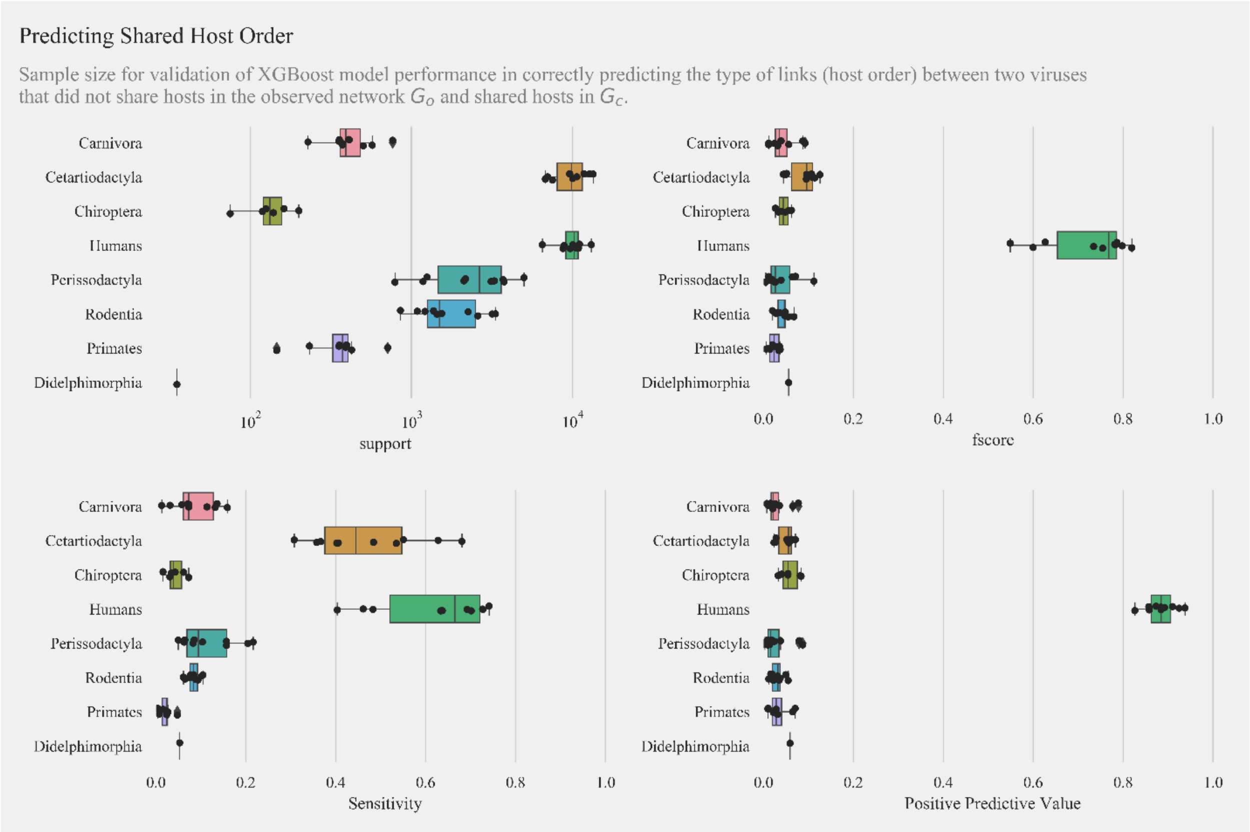

Fig. S 9. Sample size, f-score, sensitivity, and positive predictive value for validation of multiclass multioutput XGBoost model for correctly predicting the type of host sharing between viruses that did not share hosts in $\boldsymbol{G_o}$ and shared hosts in $\boldsymbol{G_c}$.

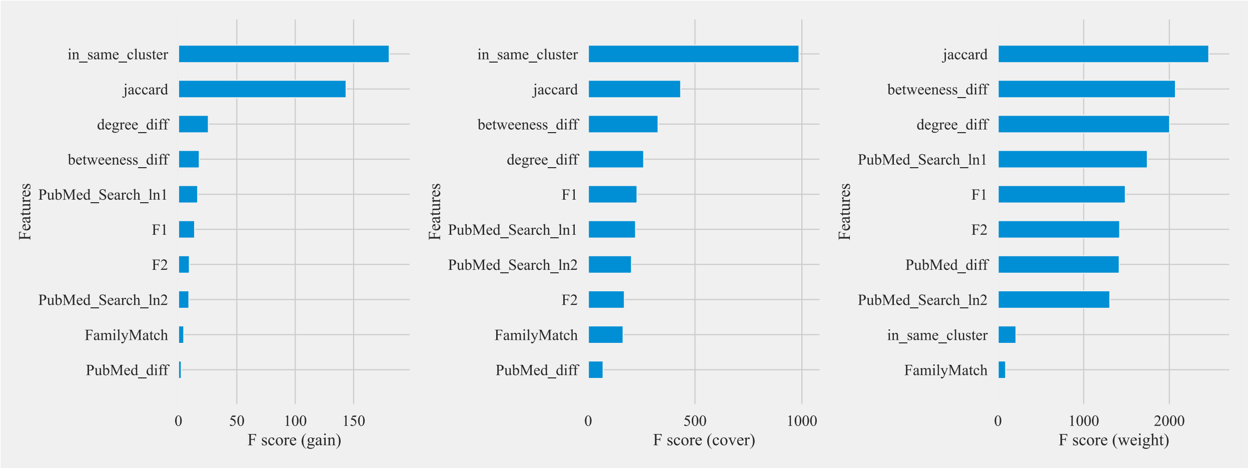

Fig. S 10: Feature importance of network topological characteristics and virological features used in training binary model based on gain, cover and weight.

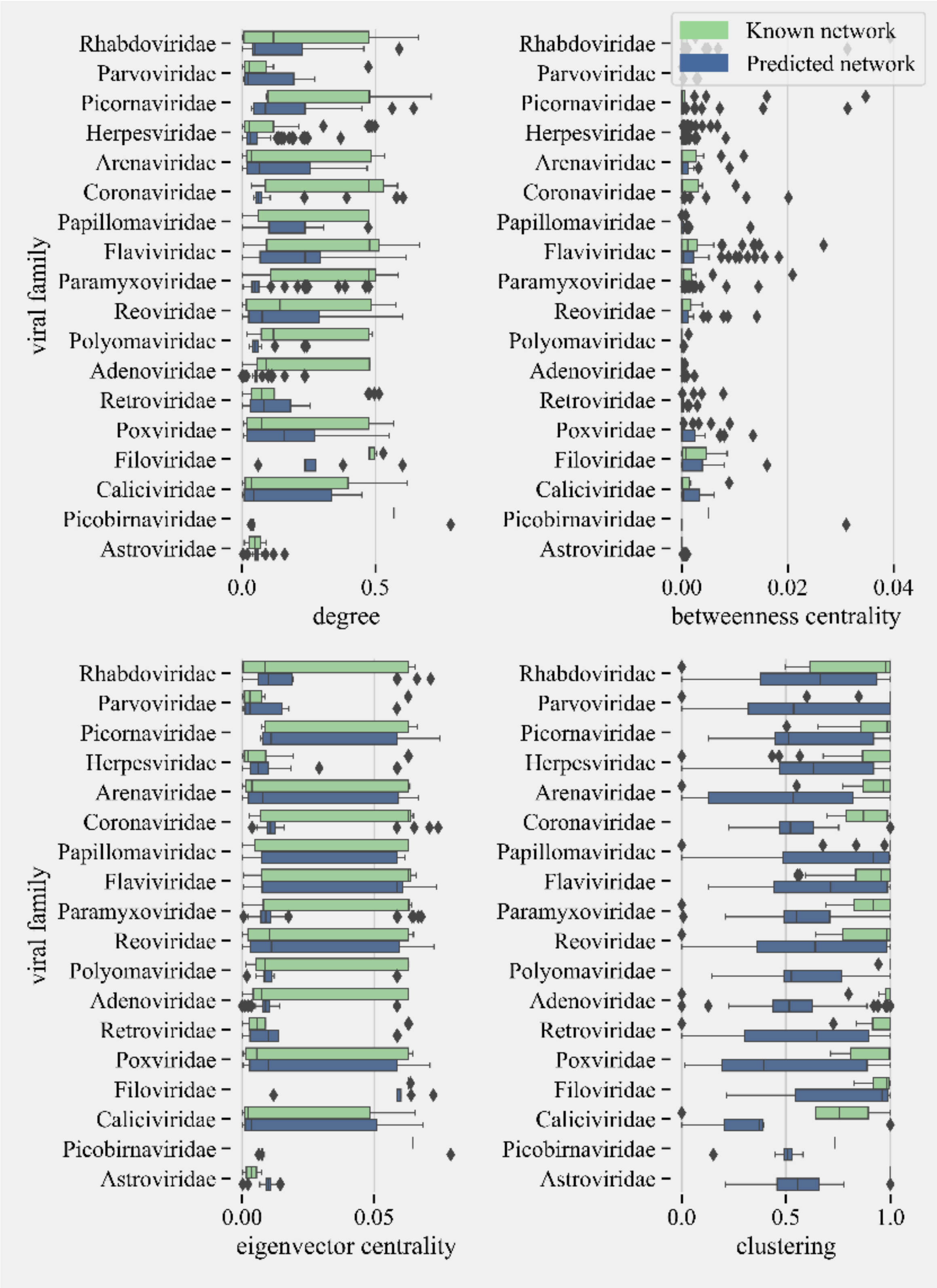

Fig. S 11: Comparison of centrality measures and clustering coefficient for selected viral families in predicted and known network.

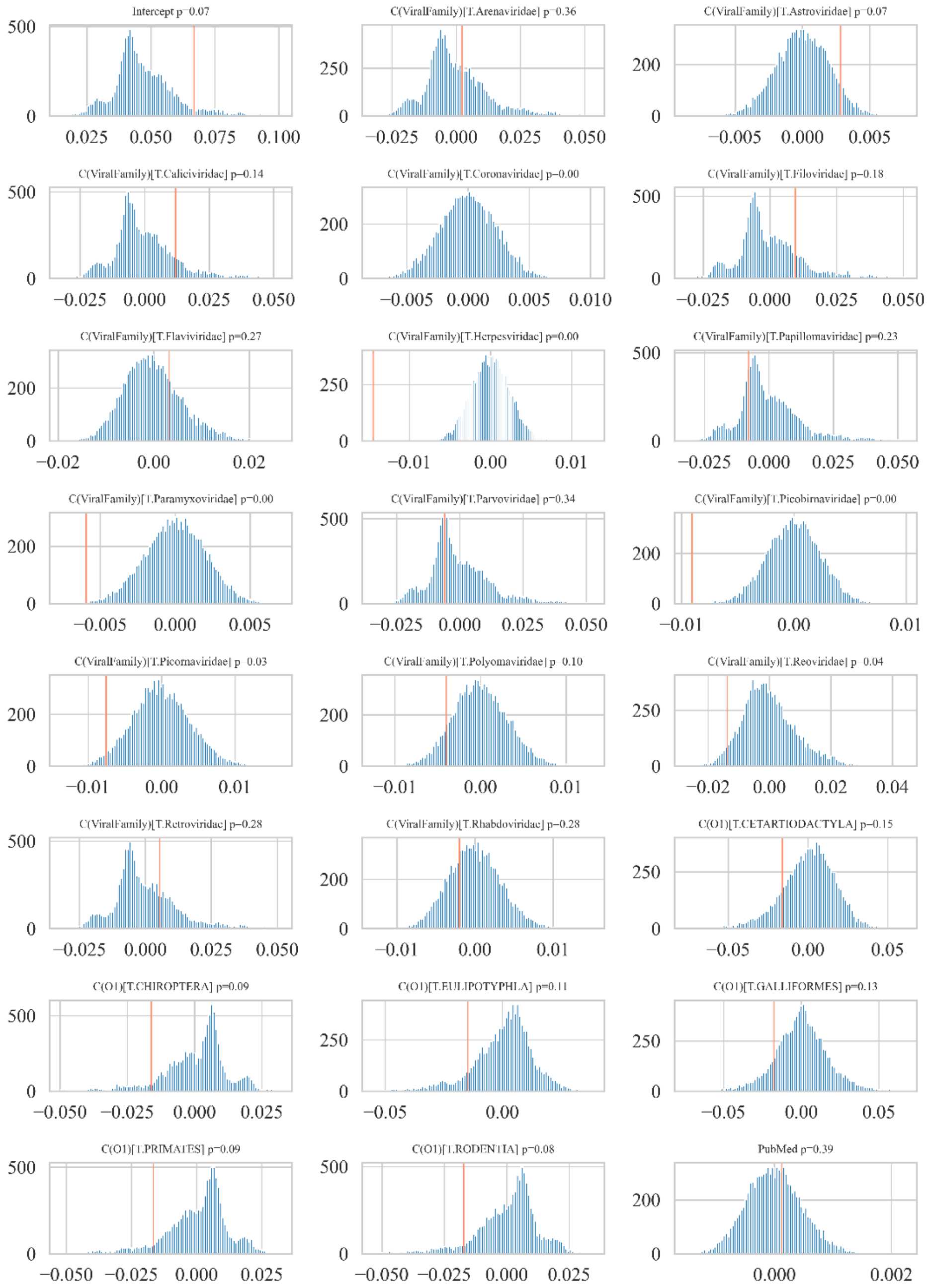

Fig. S 12: Results of permutation-based regression analysis to understand the effects of virus family, host taxonomic order and number of detections on the degree centrality of novel viruses in the predicted network ($\boldsymbol{G_{predicted}}$). Blue lines show the distribution of coefficients after 10,000 random permutations. Red lines show original coefficients.

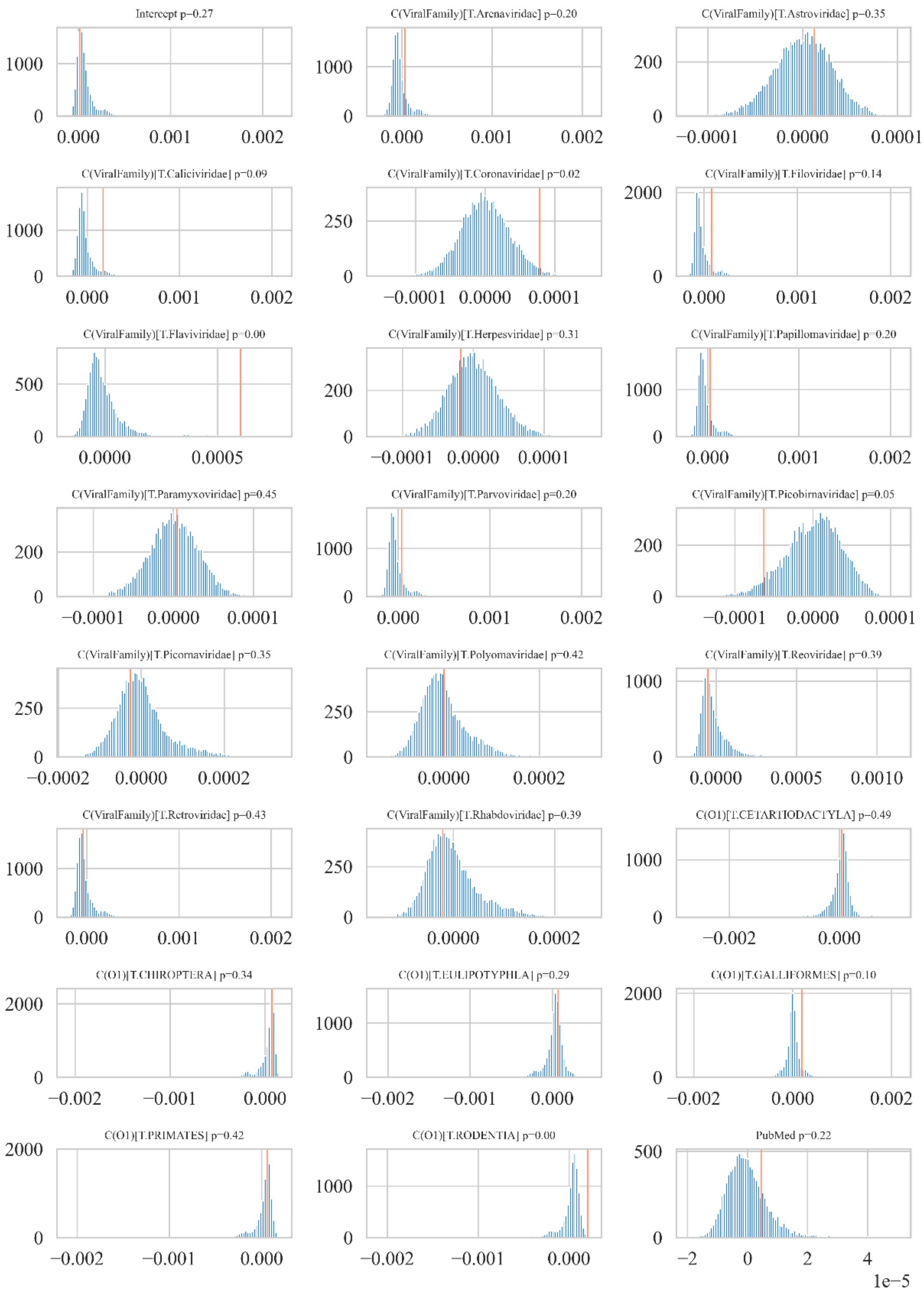

Fig. S 13: Results of permutation-based regression analysis to understand the effects of virus family, host taxonomic order, and number of detections on the betweenness centrality of novel viruses in the predicted network ($\boldsymbol{G_{predicted}}$). Blue lines show the distribution of coefficients after 10,000 random permutations. Red lines show original coefficients.

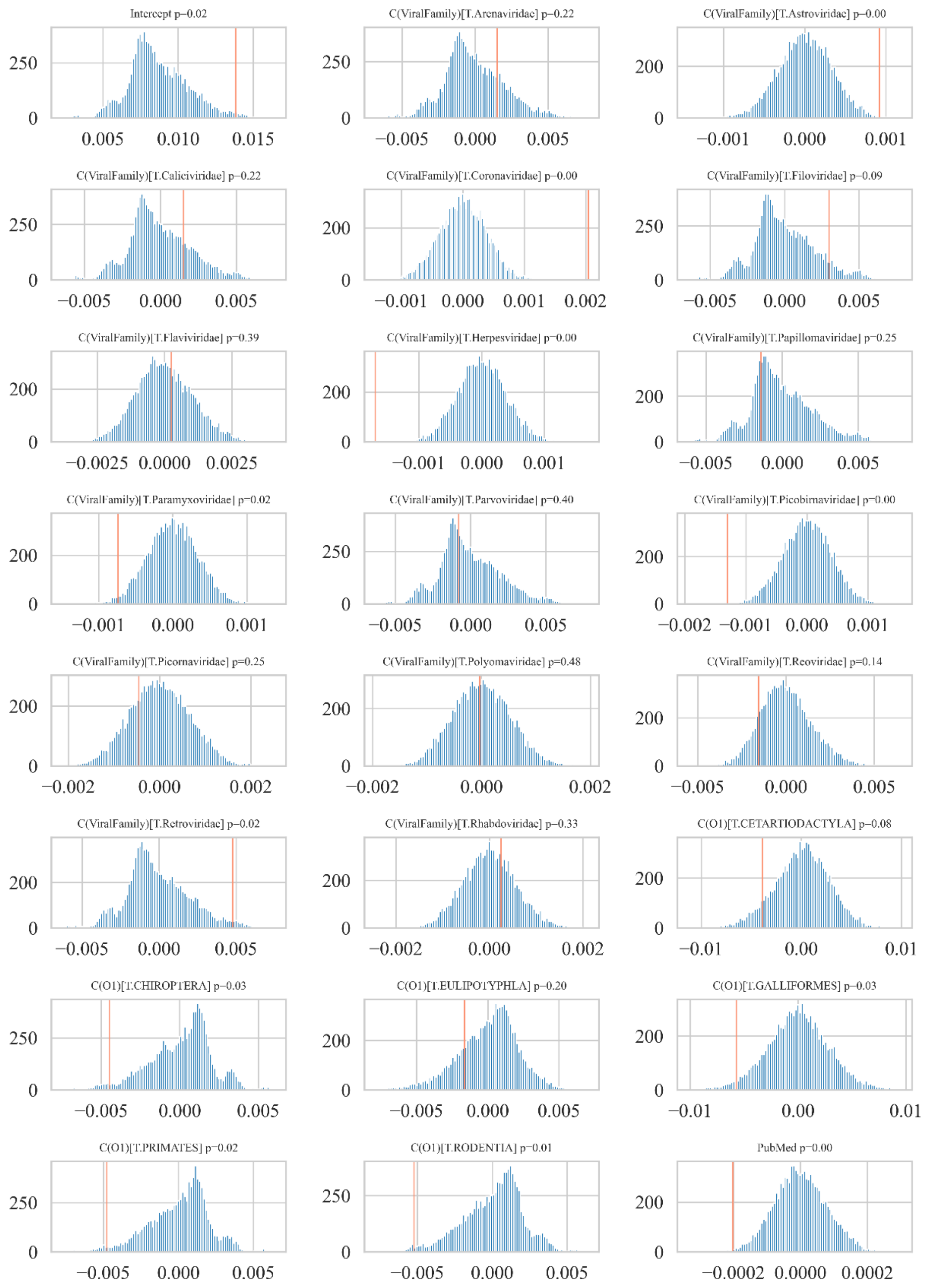

Fig. S 14: Results of permutation-based regression analysis to understand the effects of virus family, host taxonomic order and number of detections on the eigenvector centrality of novel viruses in the predicted network ($G_{predicted}$). Blue lines show the distribution of coefficients after 10,000 random permutations. Red lines show original coefficients.

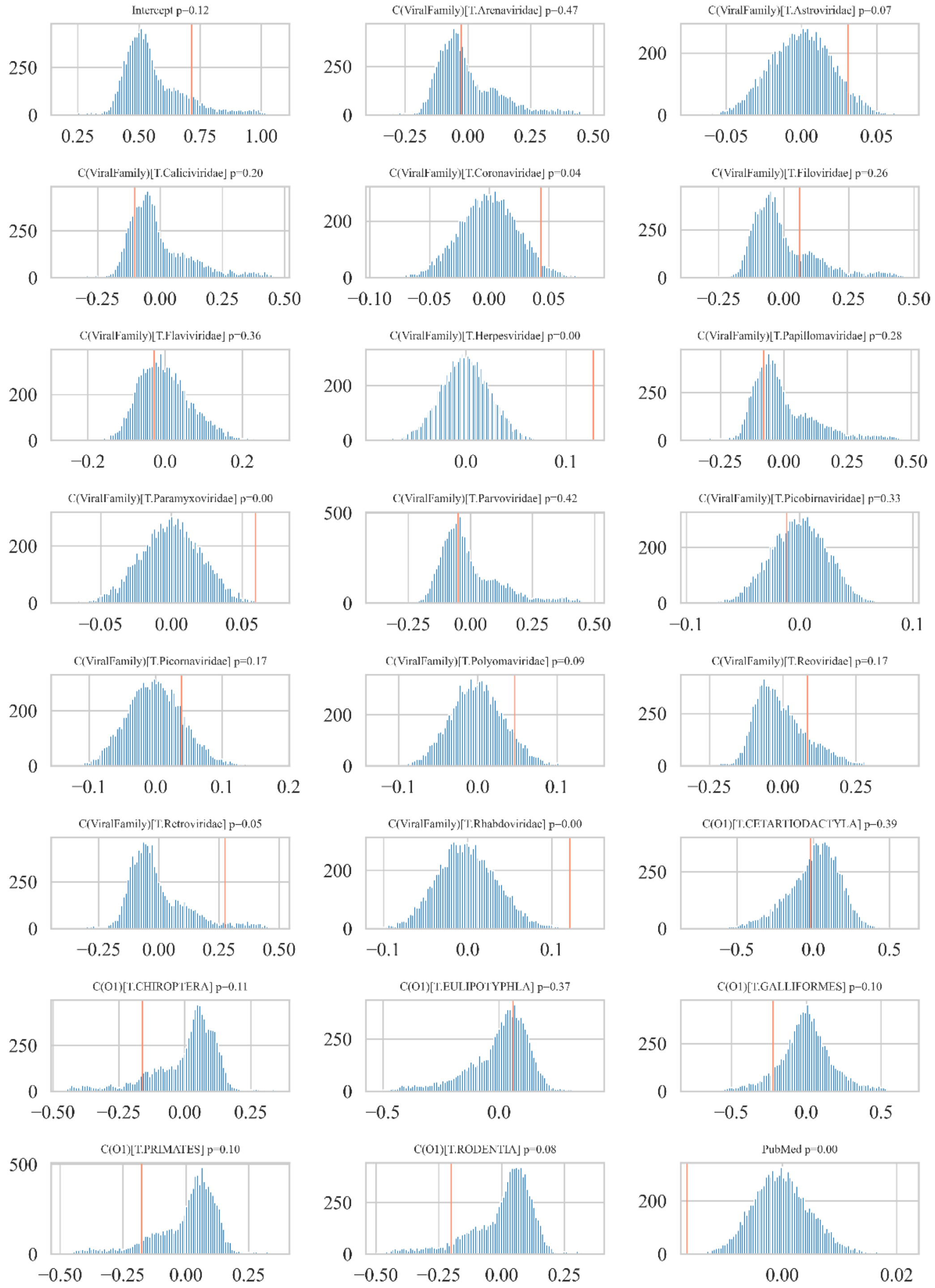

Fig. S 15: Results of permutation-based regression analysis to understand the effects of virus family, host taxonomic order and number of detections on the clustering coefficient of novel viruses in the predicted network ($\boldsymbol{G_{predicted}}$). Blue lines show the distribution of coefficients after 10,000 random permutations. Red lines show original coefficients.

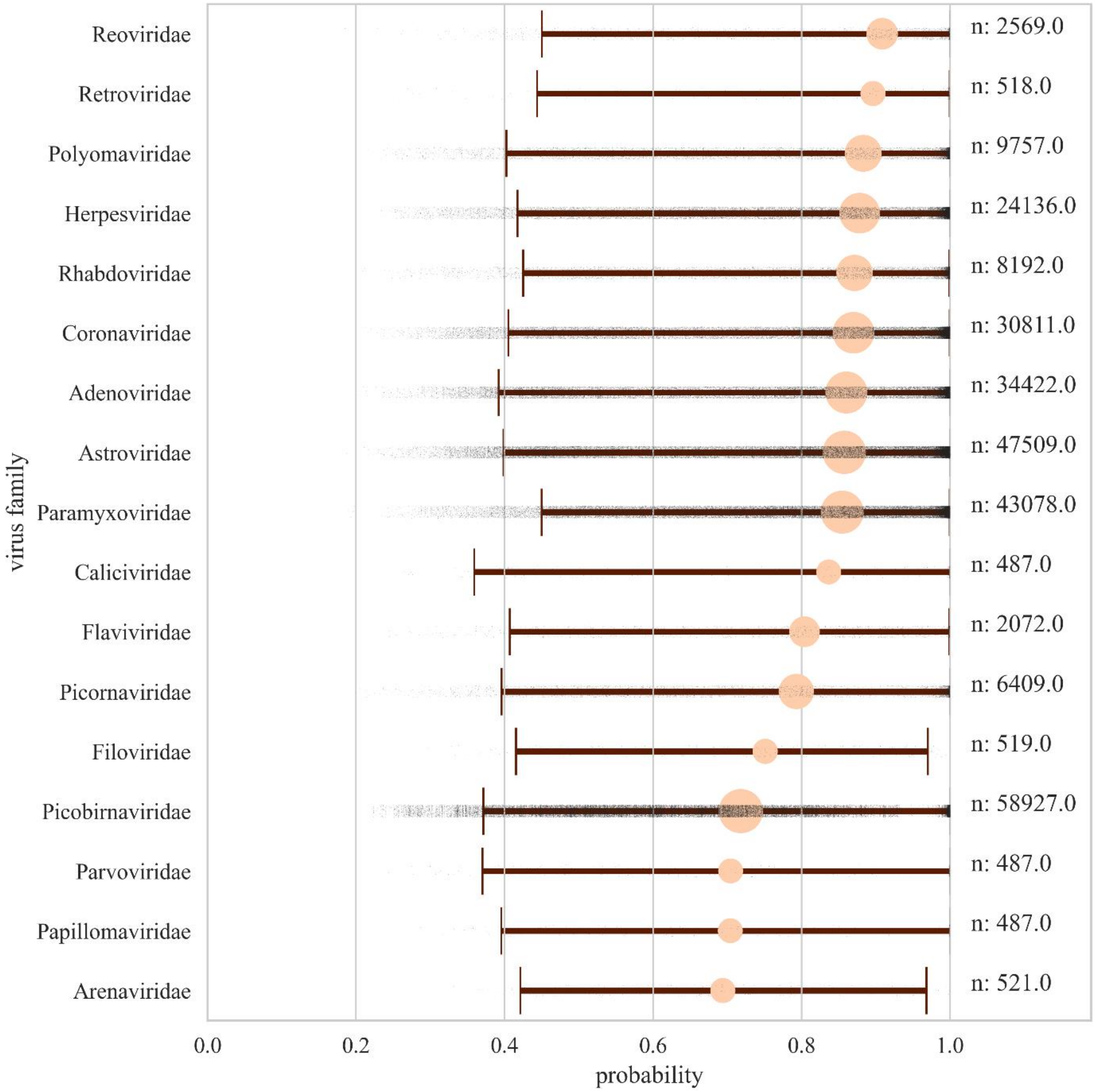

Fig. S 16: Predicted probabilities of human links between newly discovered viruses and known zoonotic and non-zoonotic viruses based on the multiclass model. Circles represent the median for all probabilities estimated for viruses within the virus family. The size of the circle is proportional to the number of human links predicted for newly discovered viruses within the virus family (represented with n). Error represents the 5th, and 95th percentile for the distribution, and grey shaded dots represent probabilities for each predicted human link.

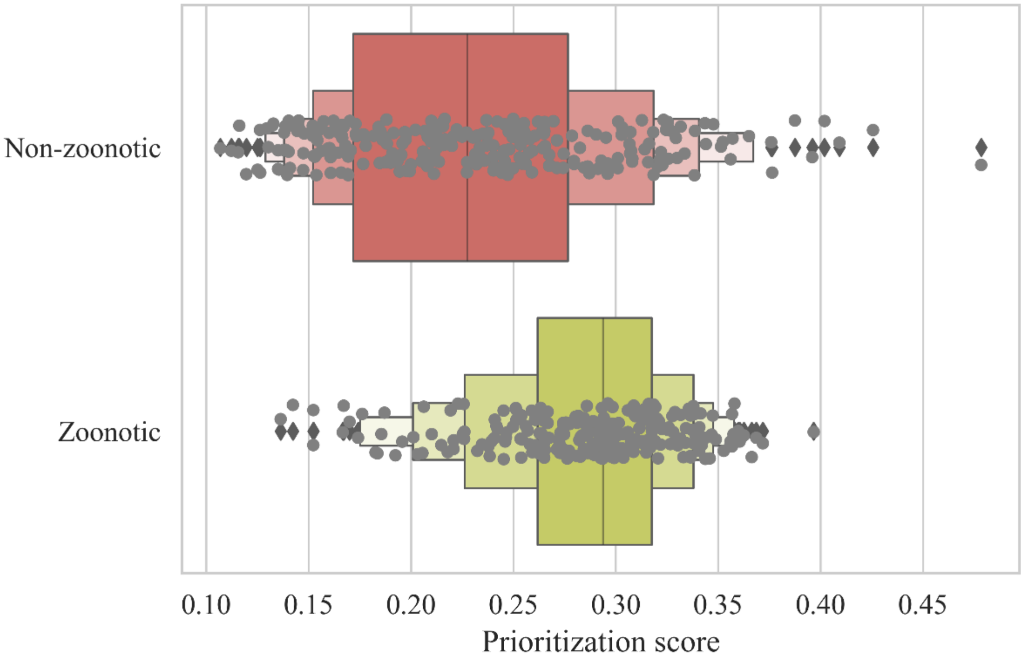

Fig. S 17: Prioritization scores for known zoonotic and non-zoonotic viruses.

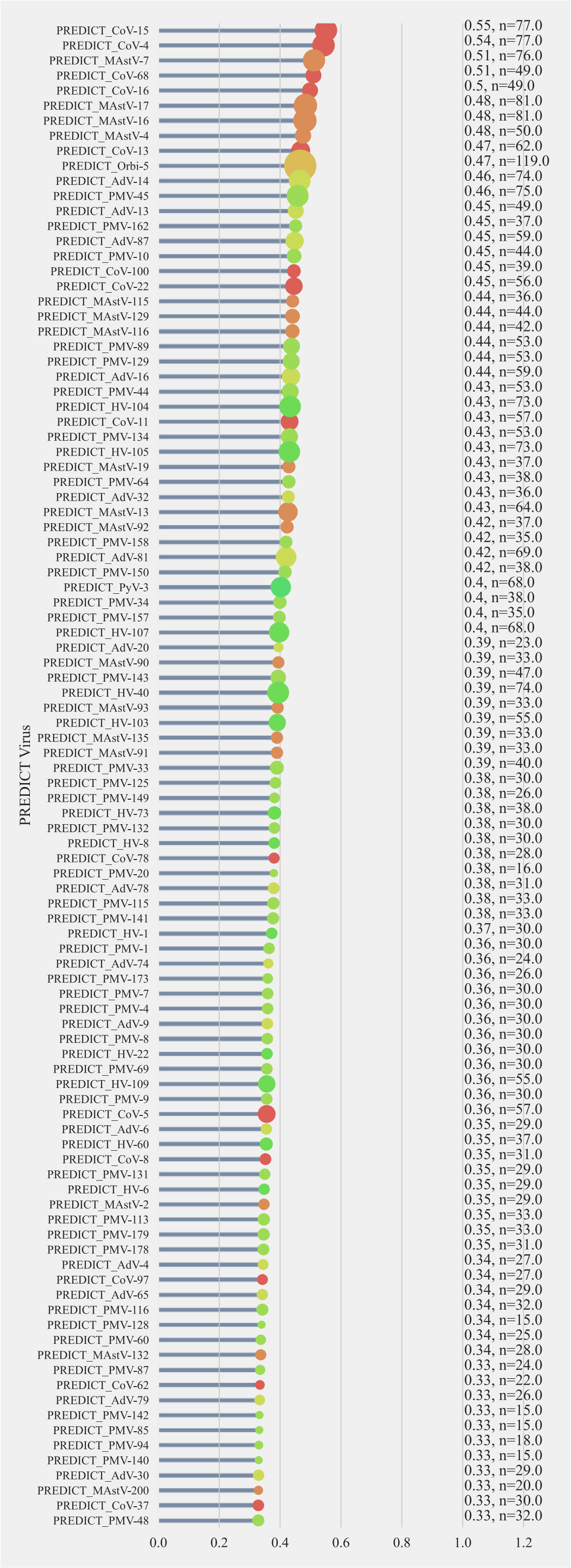

Fig. S 18: Prioritization metrics for novel viruses to understand zoonotic risk: Virus prioritization scores based on multiclass model predictions. Annotations show the score and support represented by number of human links predicted (Part 1).

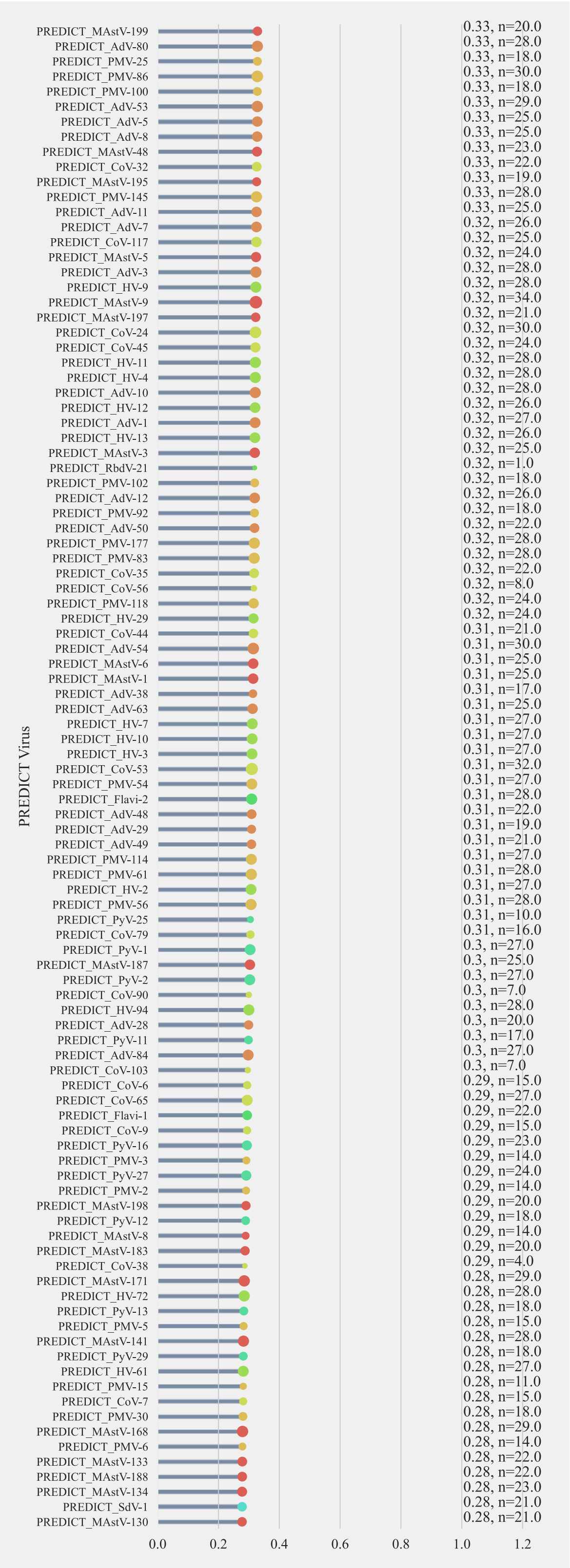

Fig. S 19: Prioritization metrics for novel viruses to understand zoonotic risk: Virus prioritization scores based on multiclass model predictions. Annotations show the score and support represented by number of human links predicted (Part 2).

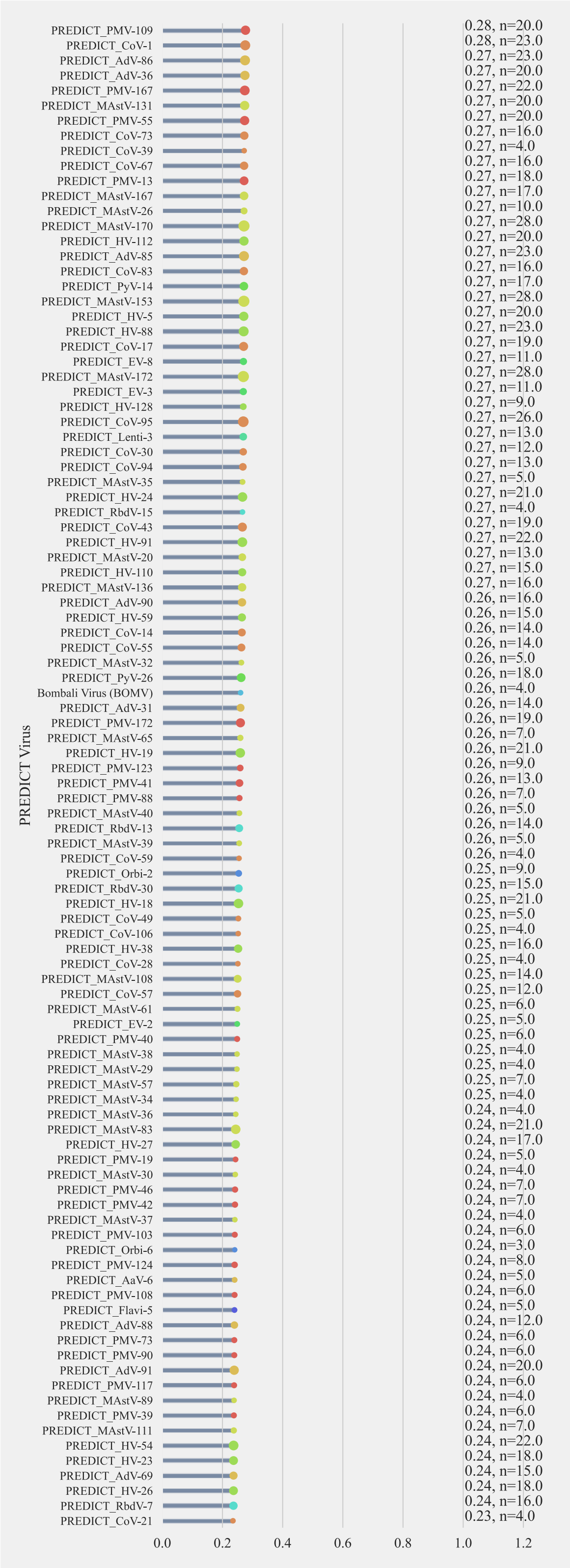

Fig. S 20: Prioritization metrics for novel viruses to understand zoonotic risk: Virus prioritization scores based on multiclass model predictions. Annotations show the score and support represented by number of human links predicted (Part 3).

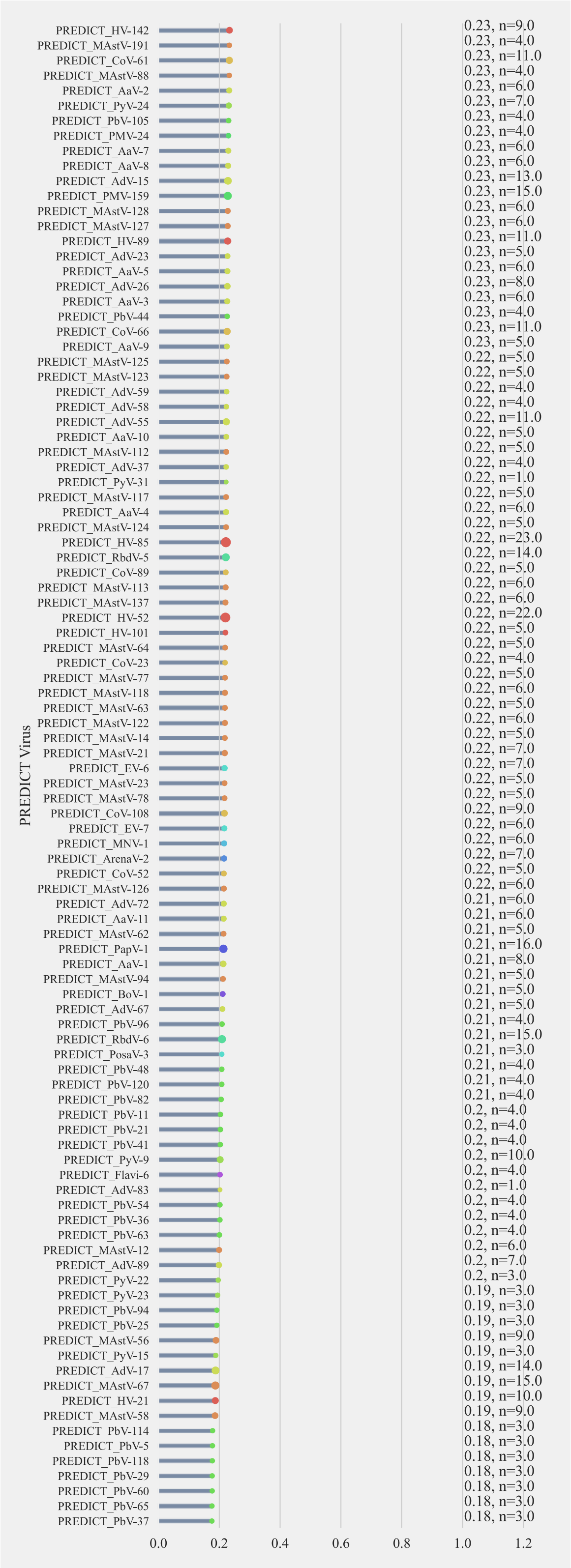

Fig. S 21: Prioritization metrics for novel viruses to understand zoonotic risk: Virus prioritization scores based on multiclass model predictions. Annotations show the score and support represented by number of human links predicted (Part 4).

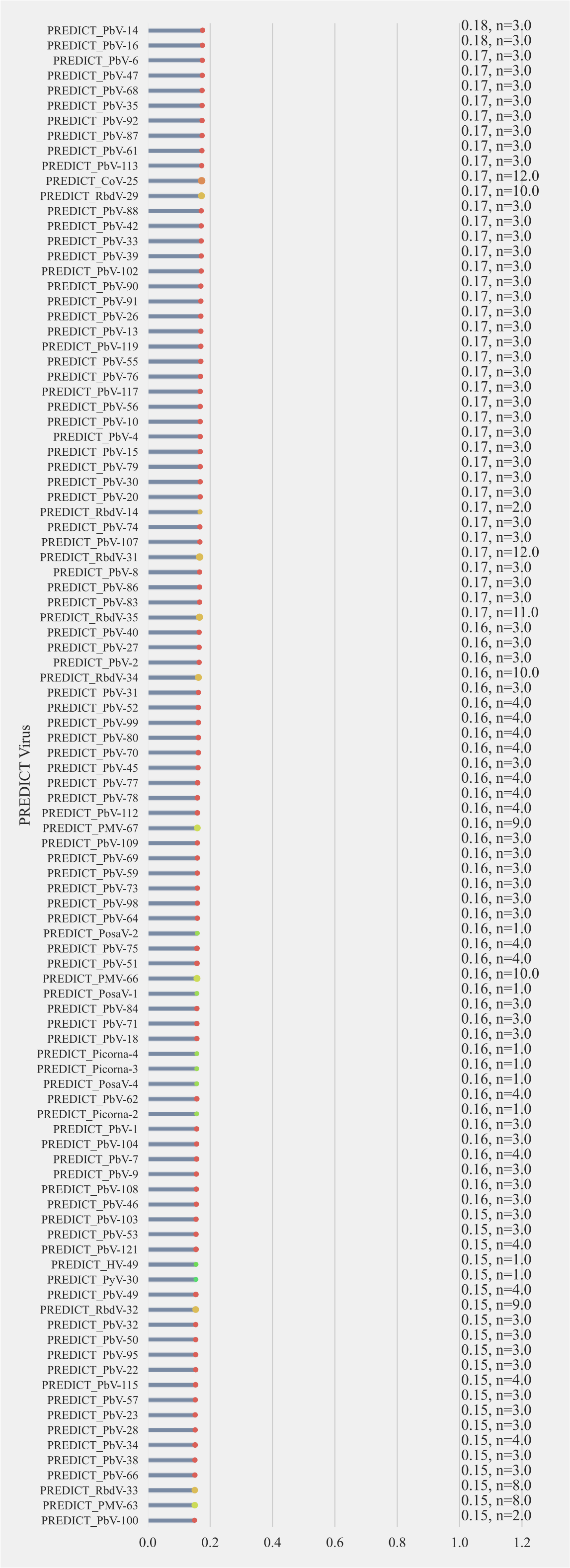

Fig. S 22: Prioritization metrics for novel viruses to understand zoonotic risk: Virus prioritization scores based on multiclass model predictions. Annotations show the score and support represented by number of human links predicted (Part 5)

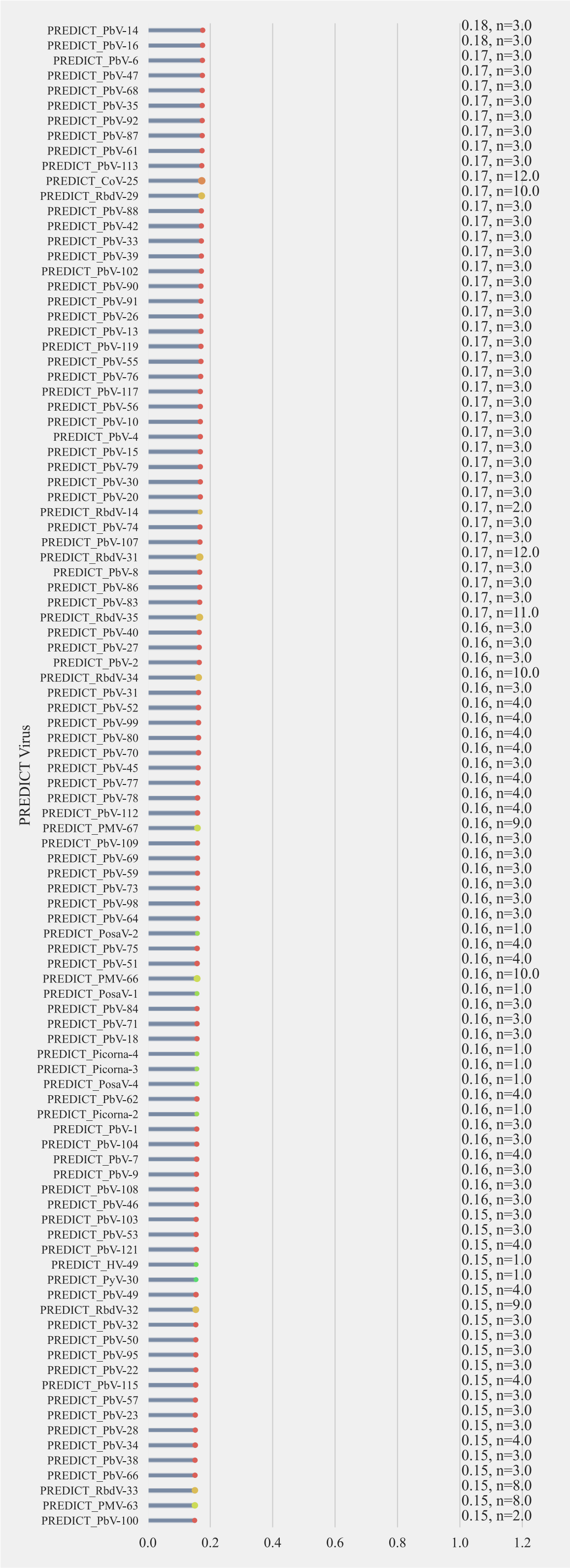

Fig. S 23: Prioritization metrics for novel viruses to understand zoonotic risk: Virus prioritization scores based on multiclass model predictions. Annotations show the score and support represented by number of human links predicted (Part 6)

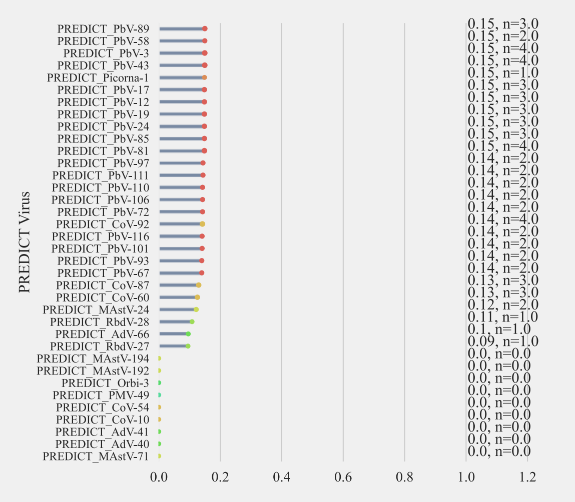

Fig. S 24: : Prioritization metrics for novel viruses to understand zoonotic risk: Virus prioritization scores based on multiclass model predictions. Annotations show the score and support represented by number of human links predicted (Part 7).

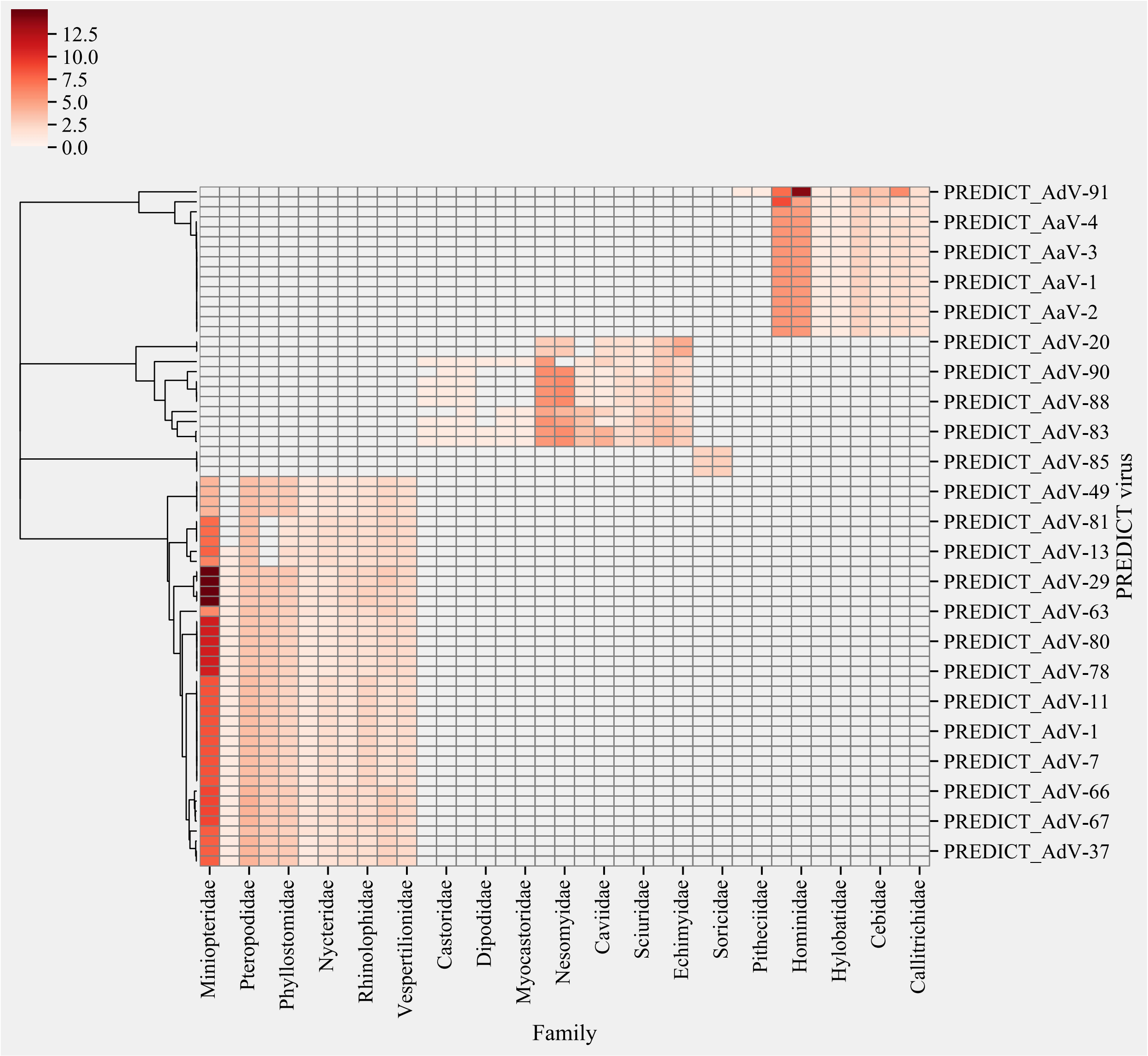

Fig. S 25: Surveillance targets for PREDICT adenoviruses based on predicted sharing of hosts with known viruses than their hosts. Red color represents the evidence towards species in the taxonomic family (frequency of links and probability). Clustering is based on Bray-curtis dissimilarity index.

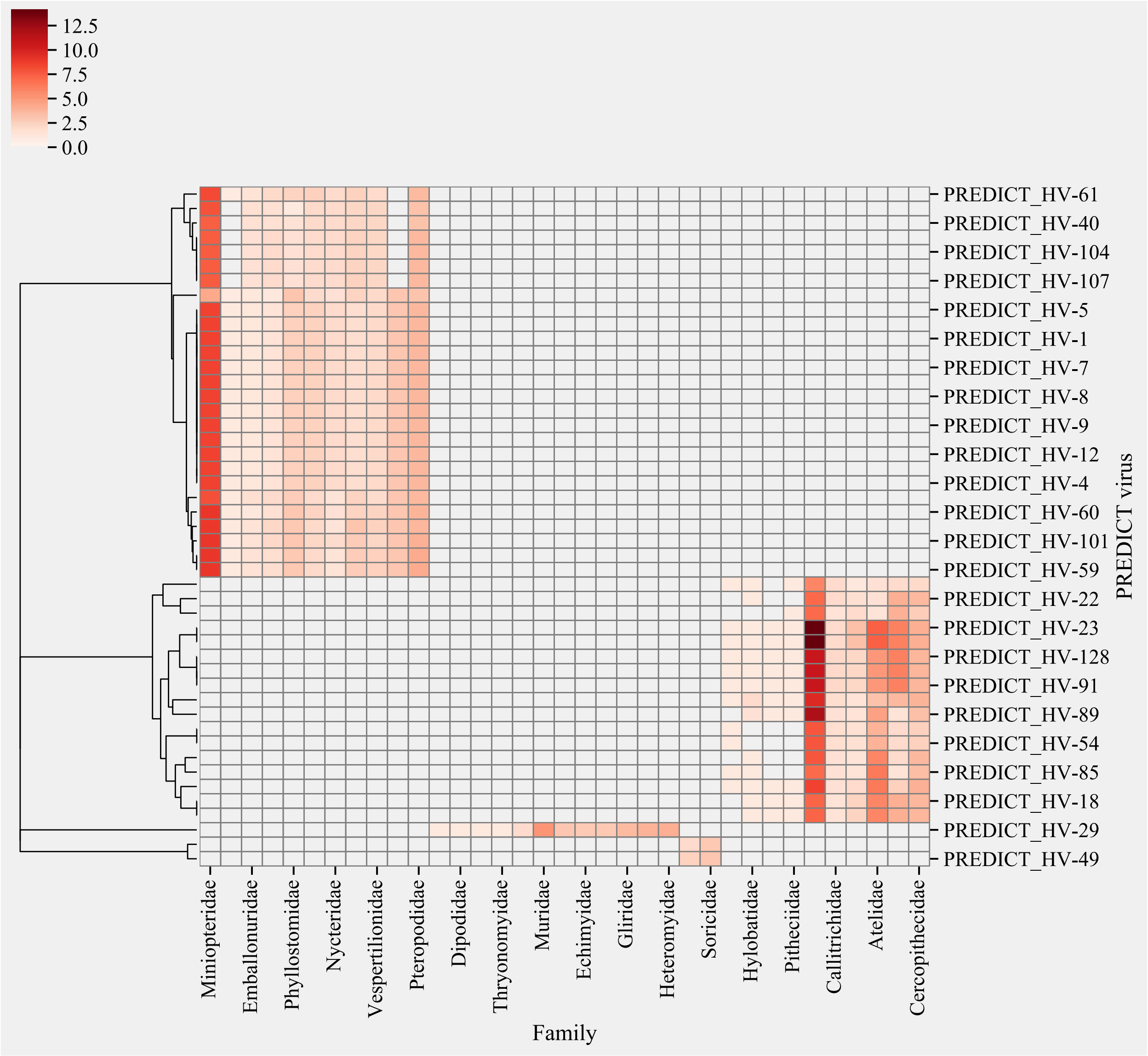

Fig. S 26: Surveillance targets for PREDICT herpesviruses based on predicted sharing of hosts with known viruses than their hosts. Red color represents the evidence towards species in the taxonomic family (frequency of links and probability). Clustering is based on Bray-curtis dissimilarity index.

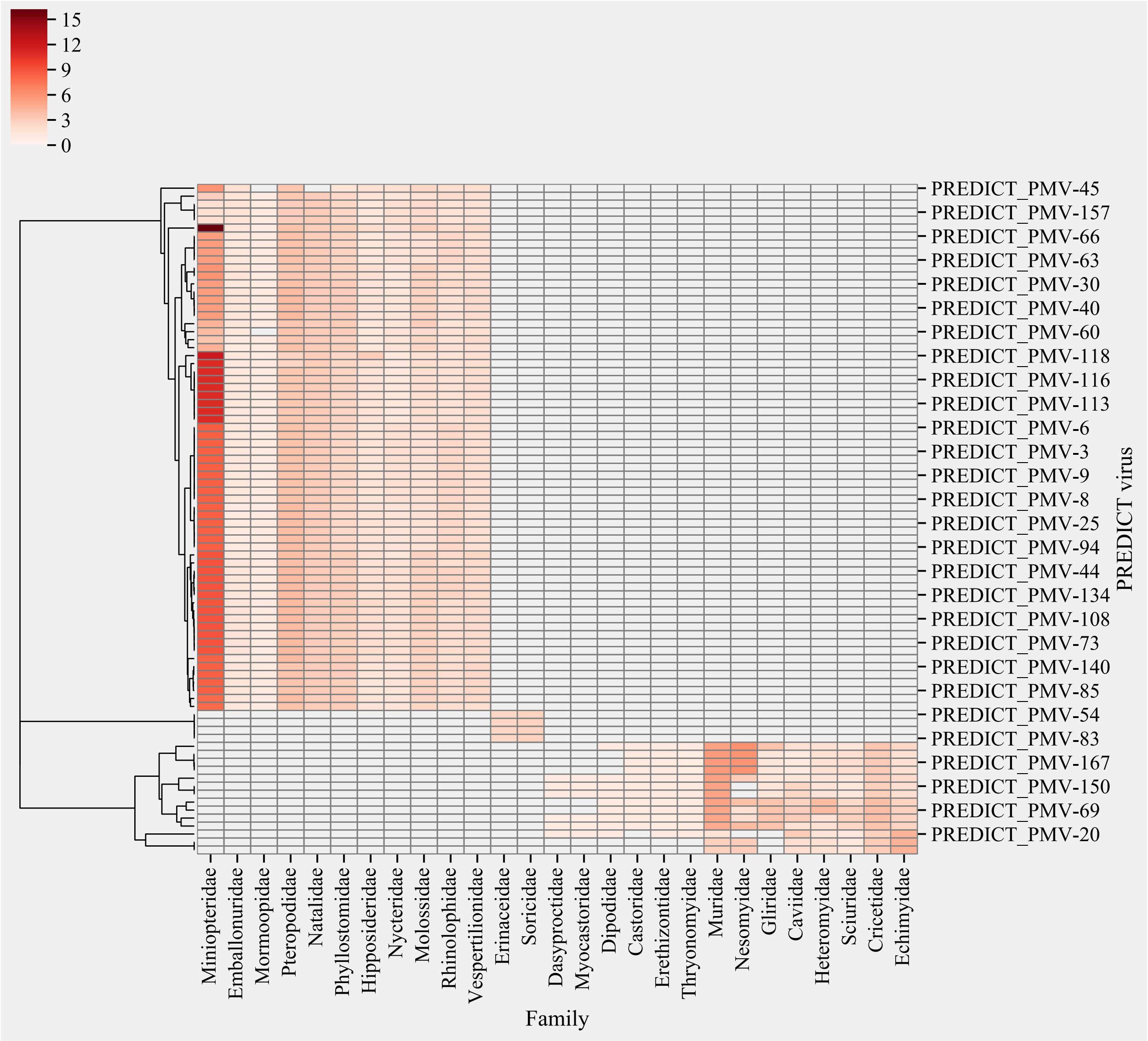

Fig. S 27: Surveillance targets for PREDICT paramyxoviruses based on predicted sharing of hosts with known viruses than their hosts. Red color represents the evidence towards species in the taxonomic family (frequency of links and probability). Clustering is based on Bray-curtis dissimilarity index.

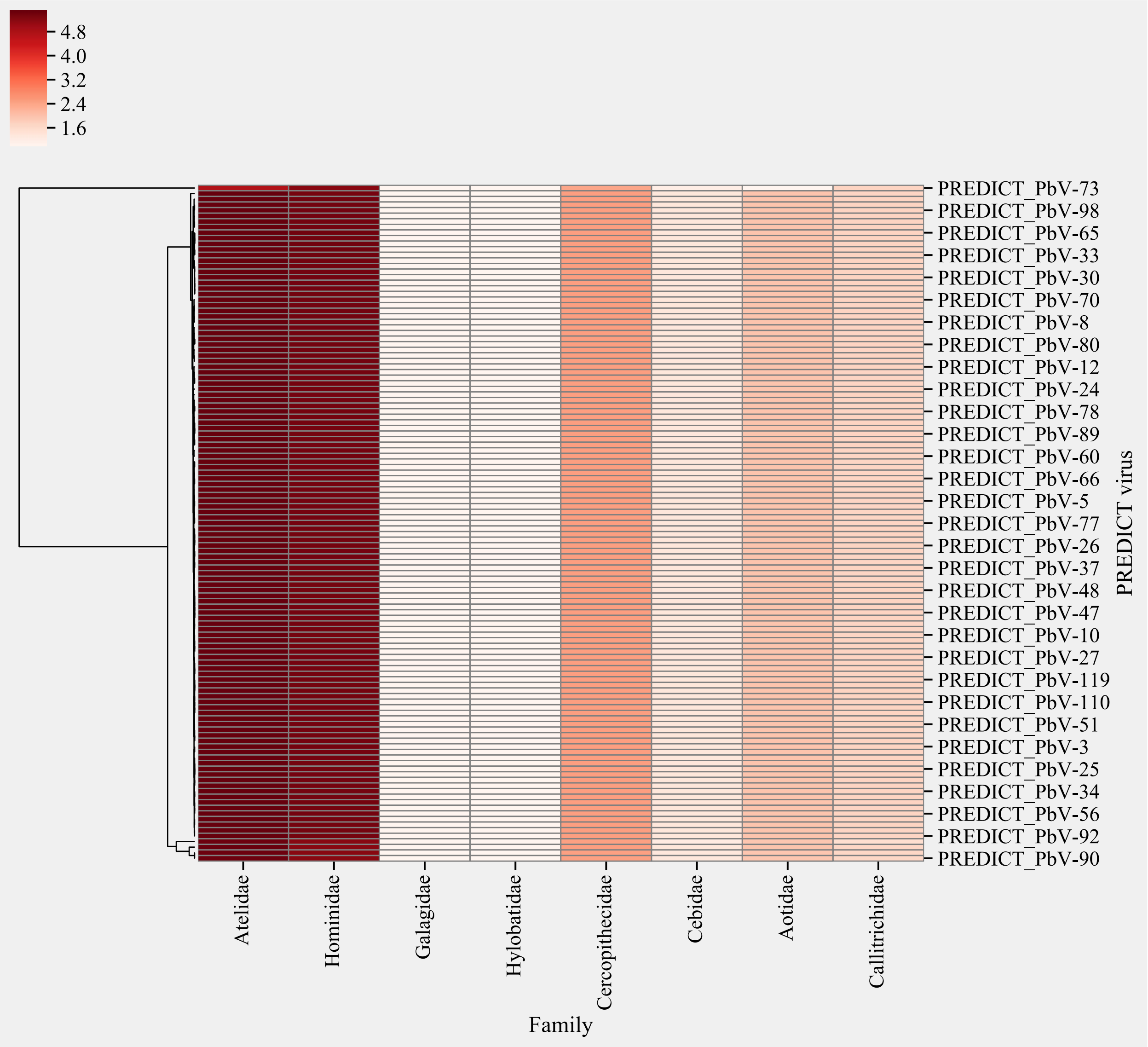

Fig. S 28: Surveillance targets for PREDICT picobirnaviruses based on predicted sharing of hosts with known viruses than their hosts. Red color represents the evidence towards species in the taxonomic family (frequency of links and probability). Clustering is based on Bray-curtis dissimilarity index.

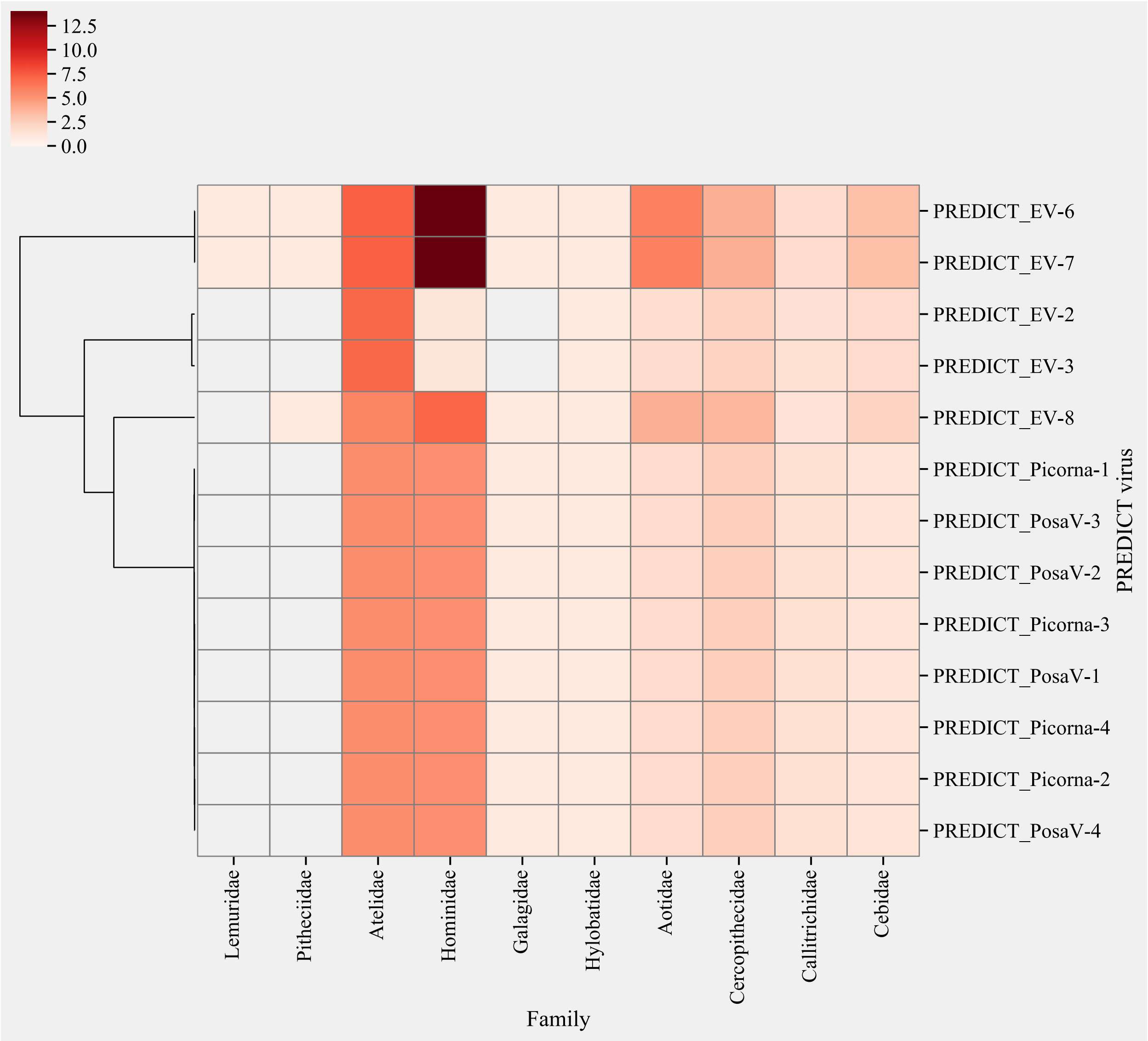

Fig. S 29: Surveillance targets for PREDICT picornaviruses based on predicted sharing of hosts with known viruses than their hosts. Red color represents the evidence towards species in the taxonomic family (frequency of links and probability). Clustering is based on Bray-curtis dissimilarity index.

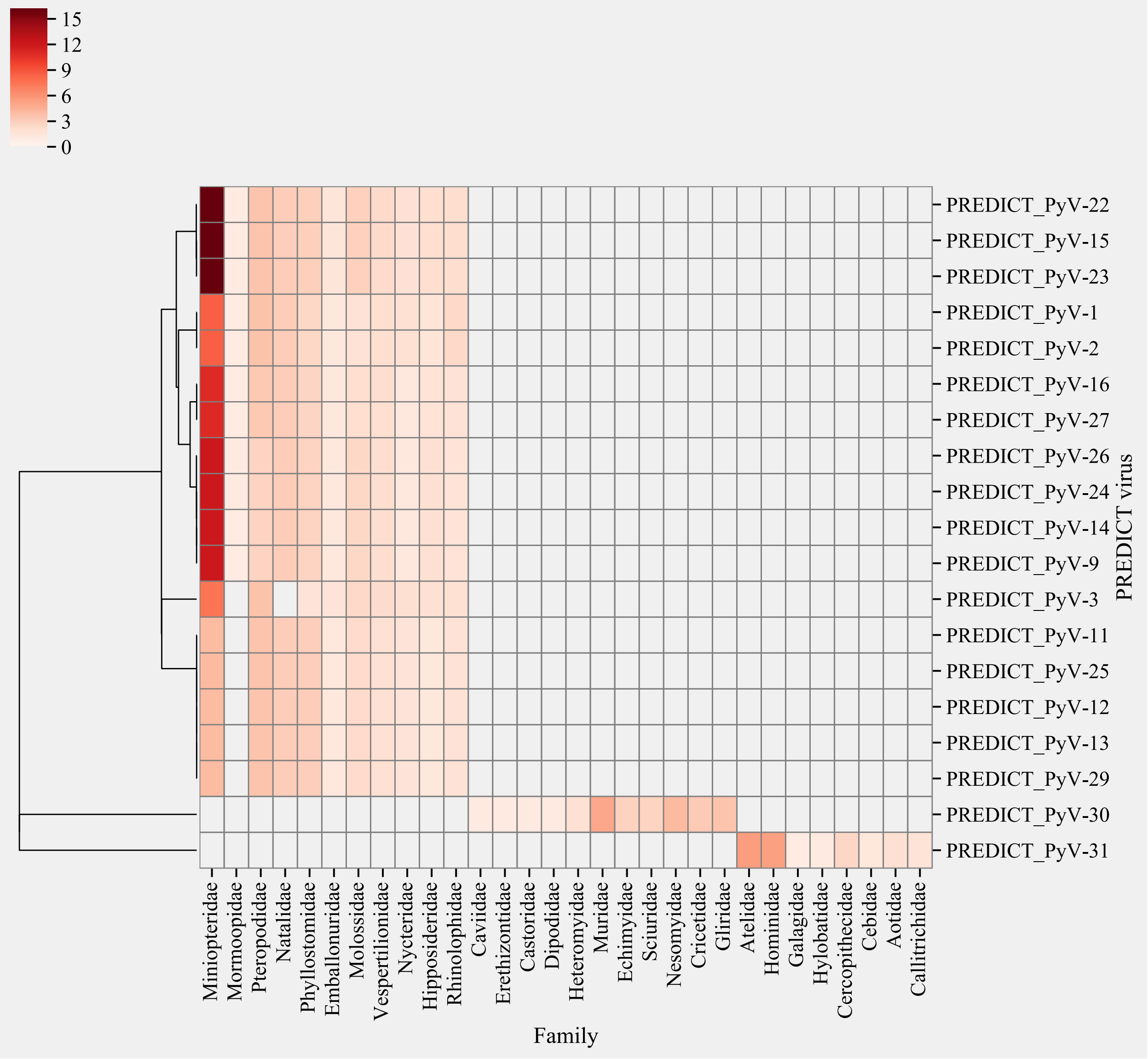

Fig. S 30: Surveillance targets for PREDICT polyomaviruses based on predicted sharing of hosts with known viruses than their hosts. Red color represents the evidence towards species in the taxonomic family (frequency of links and probability). Clustering is based on Bray-curtis dissimilarity index.

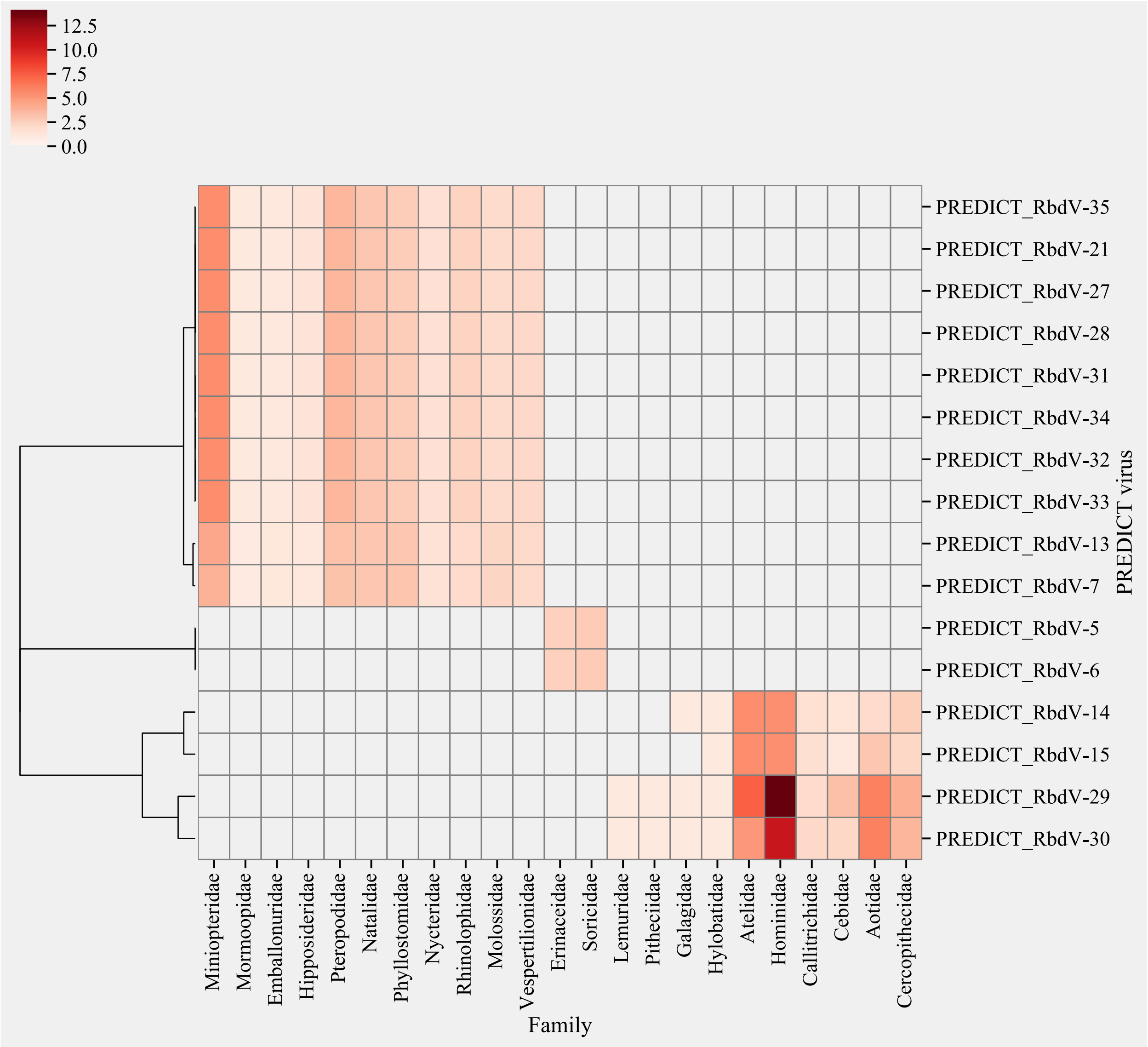

Fig. S 31: Surveillance targets for PREDICT rhabdoviruses based on predicted sharing of hosts with known viruses than their hosts. Red color represents the evidence towards species in the taxonomic family (frequency of links and probability). Clustering is based on Bray-curtis dissimilarity index.

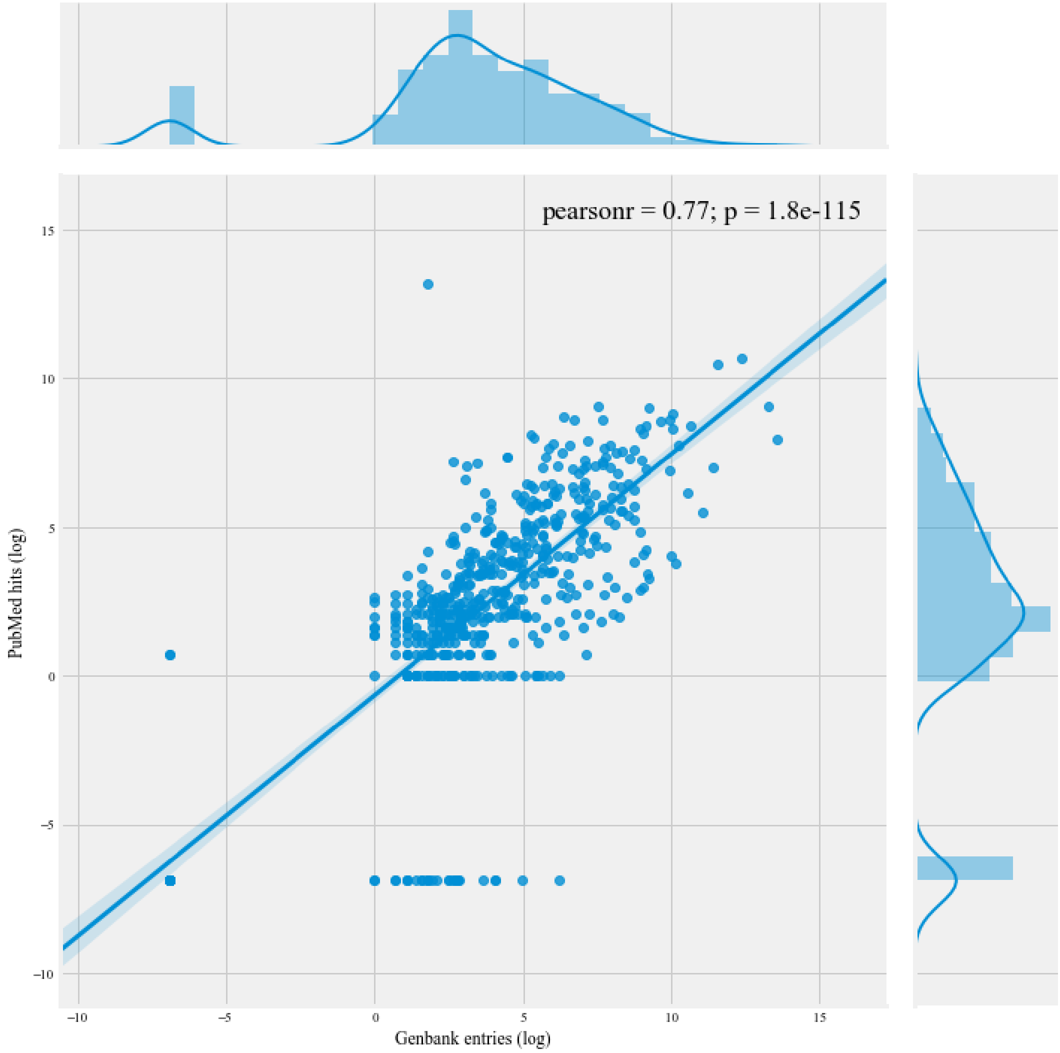

Fig. S 32: Pearson Correlation between PubMed hits (PMH1) and GenBank Entries (PMH2) for individual viruses in the known network. Marginal plots show distribution of individual axes values.

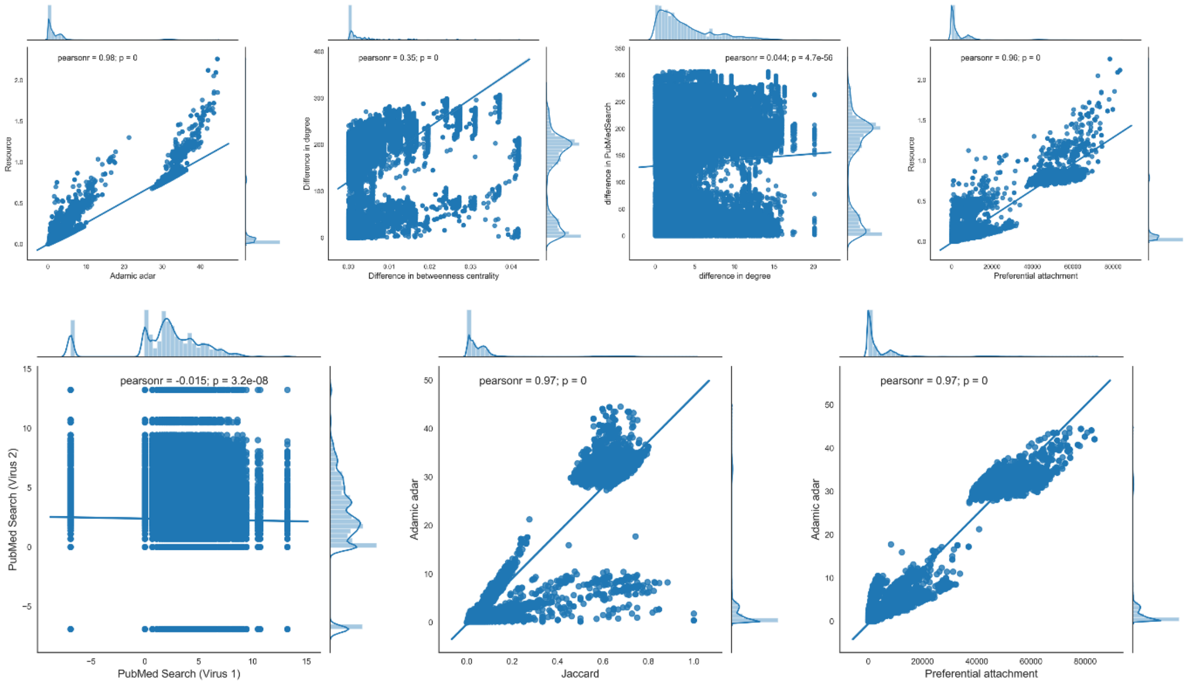

Fig. S 33: Jointplots showing correlations of network topological features for all node pairs in observed network ($\boldsymbol{G_c}$).

	Number of predicted links of novel viruses with known viruses generated due to sharing of humans as host		
Predictors	*Incidence Rate Ratios*	*CI*	*p*
(Intercept)	41.37	16.16 – 105.89	**<0.001**
Adenoviridae	*Reference*		
Arenaviridae	0.48	0.11 – 2.16	0.338
Astroviridae	0.84	0.68 – 1.04	0.113
Caliciviridae	0.79	0.17 – 3.74	0.764
Coronaviridae	0.84	0.66 – 1.07	0.167
Filoviridae	0.35	0.07 – 1.86	0.218
Flaviviridae	0.72	0.36 – 1.45	0.356
Herpesviridae	1.59	1.28 – 1.98	**<0.001**
Papillomaviridae	1.64	0.49 – 5.48	0.425
Paramyxoviridae	1.12	0.91 – 1.36	0.281
Parvoviridae	0.70	0.14 – 3.51	0.664
Picobirnaviridae	0.53	0.40 – 0.69	**<0.001**
Picornaviridae	0.46	0.27 – 0.81	**0.007**
Polyomaviridae	0.75	0.53 – 1.06	0.100
Reoviridae	0.85	0.31 – 2.28	0.741
Retroviridae	1.38	0.38 – 5.02	0.620
Rhabdoviridae	0.56	0.37 – 0.85	**0.007**
CARNIVORA	*Reference*		
CETARTIODACTYLA	0.39	0.08 – 2.00	0.258
CHIROPTERA	0.48	0.19 – 1.21	0.119
EULIPOTYPHLA	0.42	0.16 – 1.15	0.092
GALLIFORMES	1.19	0.34 – 4.25	0.784

PRIMATES	0.28	0.11 – 0.73	**0.009**
RODENTIA	0.52	0.20 – 1.34	0.174
PubMed	1.03	0.98 – 1.08	0.240
Observations	535		

Table S 1: Negative binomial Generalized linear mixed model to understand association between virus family, taxonomic order of the host and PubMed Hists on the predicted number of links of novel viruses generated with known viruses formed due to sharing of humans as hosts **Number of human links ~ Virus Family + host Taxonomic Order + PubMed hits, family = negative binomial**

	Mean probability of virus links formed due to humans as hosts		
Predictors	*Risk Ratios*	*CI*	*p*
(Intercept)	3.10	1.28 – 9.06	**0.020**
Adenoviridae	*Reference*		
Arenaviridae	0.61	0.28 – 1.32	0.198
Astroviridae	0.98	0.87 – 1.12	0.809
Caliciviridae	0.91	0.43 – 1.97	0.799
Coronaviridae	1.00	0.86 – 1.17	0.949
Filoviridae	1.36	0.59 – 3.51	0.492
Flaviviridae	0.98	0.66 – 1.48	0.923
Herpesviridae	1.07	0.92 – 1.25	0.393
Papillomaviridae	0.79	0.37 – 1.69	0.539
Paramyxoviridae	1.17	1.02 – 1.33	**0.026**
Parvoviridae	0.92	0.43 – 2.01	0.831
Picobirnaviridae	0.79	0.68 – 0.92	**0.002**
Picornaviridae	1.14	0.89 – 1.47	0.314
Polyomaviridae	1.03	0.84 – 1.27	0.786
Reoviridae	1.34	0.75 – 2.50	0.333
Retroviridae	1.39	0.63 – 3.32	0.435
Rhabdoviridae	0.69	0.56 – 0.85	**0.001**
CARNIVORA	*Reference*		
CETARTIODACTYLA	0.21	0.06 – 0.67	**0.011**
CHIROPTERA	0.58	0.20 – 1.40	0.268
EULIPOTYPHLA	0.53	0.18 – 1.33	0.211
GALLIFORMES	1.26	0.30 – 5.77	0.753
PRIMATES	0.47	0.16 – 1.13	0.121

RODENTIA	0.66	0.23 – 1.61	0.402
PubMed	1.02	0.98 – 1.05	0.346
Observations	535		

Table S 2: Quasibinomial Generalized linear mixed model to understand association between virus family, taxonomic order of the host and PubMed Hists on the predicted mean probability of sharing a human as a link with another virus.

probability of link being human ~ Virus Family + host Taxonomic Order + PubMed hits, family = quasibinomial

Feature	Calculation/explanation	Variable type
Jaccard coefficient	$\frac{\lvert\Gamma(virus_1) \cap \Gamma(virus_2)\rvert}{\lvert\Gamma(virus_1) \cup \Gamma(virus_2)\rvert}$ where $\Gamma(virus)$ denotes set of neighbors of $virus$	Numeric
The difference in degree centrality	The absolute difference in the number of connections (edges) of viruses in the virus pair.	Numeric
The difference in betweenness centrality	Betweenness centrality of a node ($virus$) is the sum of the fraction of all-pairs shortest paths that pass through $virus$ and is calculated as $c_B(virus) = \sum_{s,t \in viruses} \frac{\sigma(s,t\mid virus)}{\sigma(s,t)}$ The difference was calculated as $\lvert c_B(virus_1) - c_B(virus_2)\rvert$	Numeric
Same cluster	Modularity partition was estimated using Louvain clustering algorithm to check if both the viruses in the pair fall under the same modularity/cluster	Binary (1, 0)
Viral family virus 1	Taxonomical family of virus 1 in the pair	Categorical
Viral family virus 2	Taxonomical family of virus 2 in the pair	Categorical
Same viral families	A binary variable depicting if viruses in the pair belong to the same taxonomical family.	Binary (1, 0)
Difference in PubMedHits	The absolute difference in the number of sequences of both viruses in a pair detected in PubMed	Numeric
PubMedHits virus 1 (log)	Number of nucleotide sequences in PubMed for virus 1 in the pair	Numeric
PubMedHits virus 2 (log)	Number of nucleotide sequences in PubMed for virus 2 in the pair	Numeric

Table S 3. Network features, their definitions, and type of feature used in the training of GBM models.

Feature	Calculation/explanation	Variable type
Jaccard coefficient	$\frac{\|\Gamma(virus_1) \cap \Gamma(virus_2)\|}{\|\Gamma(virus_1) \cup \Gamma(virus_2)\|}$ where $\Gamma(virus)$ denotes set of neighbors of $virus$	Numeric
The difference in degree centrality	The absolute difference in the number of connections (edges) of viruses in the virus pair.	Numeric
The difference in betweenness centrality	Betweenness centrality of a node ($virus$) is the sum of the fraction of all-pairs shortest paths that pass through $virus$ and is calculated as $c_B(virus) = \sum_{s,t \in viruses} \frac{\sigma(s,t\|virus)}{\sigma(s,t)}$ The difference was calculated as $\|c_B(virus_1) - c_B(virus_2)\|$	Numeric
Same cluster	Modularity partition was estimated using Louvain clustering algorithm to check if both the viruses in the pair fall under the same modularity/cluster	Binary (1, 0)
Viral family virus 1	Taxonomical family of virus 1 in the pair	Categorical
Viral family virus 2	Taxonomical family of virus 2 in the pair	Categorical
Same viral families	A binary variable depicting if viruses in the pair belong to the same taxonomical family.	Binary (1, 0)
Difference in PubMedHits	The absolute difference in the number of sequences of both viruses in a pair detected in PubMed	Numeric
PubMedHits virus 1 (log)	Number of nucleotide sequences in PubMed for virus 1 in the pair	Numeric
PubMedHits virus 2 (log)	Number of nucleotide sequences in PubMed for virus 2 in the pair	Numeric

Table S 4. Network features, their definitions, and type of feature used in the training of GBM models.

XGboost parameter	Parameter explanation	Parameter values tested
Learning rate	Step size shrinkage used in the update to prevents overfitting.	0.05, 0.10, 0.20, 0.30
Max depth	Maximum depth of a tree.	3, 4, 8, 12, 15
Minimum child weight	The minimum sum of instance weight (hessian) needed in a child.	1, 3, 5, 7
Gamma	Minimum loss reduction that is required to make a further partition on a leaf node of the tree. The larger gamma is, the more conservative the algorithm will be.	0.0, 0.1, 0.3, 0.5
Colsample by tree	Parameters for subsampling of columns.	0.3, 0.5 , 0.7

Table S 5: Hyper-tuning of XGboost model parameters. The parameter name and values testes in the grid search to find the best performing parameters for the model.

Country	Animal Class	No. of Animals
**Bangladesh**	**Domestic**	956
	**Wildlife**	8120
**Bolivia**	**Wildlife**	717
**Brazil**	**Wildlife**	826
**Cambodia**	**Domestic**	894
	**Wildlife**	5084
**Cameroon**	**Wildlife**	7379
**China**	**Wildlife**	4912
**DR Congo**	**Domestic**	34
	**Wildlife**	4277
**Egypt**	**Wildlife**	1003
**Ethiopia**	**Wildlife**	836
**Gabon**	**Wildlife**	3127
**Ghana**	**Wildlife**	2784
**Guinea**	**Domestic**	95
	**Wildlife**	4411
**India**	**Wildlife**	19
**Indonesia**	**Wildlife**	2956
**Ivory Coast**	**Wildlife**	776
**Jordan**	**Wildlife**	1080
**Kenya**	**Domestic**	100
	**Wildlife**	811
**Lao PDR**	**Domestic**	479
	**Wildlife**	3068
**Liberia**	**Wildlife**	3795
**Malaysia, Peninsular**	**Domestic**	670
	**Wildlife**	1962
**Malaysia, Sabah**	**Wildlife**	2672
**Mexico**	**Wildlife**	1074
**Mongolia**	**Domestic**	2
	**Wildlife**	3236
**Myanmar**	**Wildlife**	1062
**Nepal**	**Wildlife**	3228
**Peru**	**Wildlife**	425
**Republic of Congo**	**Wildlife**	999
**Rwanda**	**Wildlife**	1341
**Senegal**	**Wildlife**	874
**Sierra Leone**	**Domestic**	267
	**Wildlife**	7587
**South Sudan**	**Wildlife**	271

**Tanzania**	**Domestic**	372
	**Wildlife**	4578
**Thailand**	**Domestic**	205
	**Wildlife**	3819
**Uganda**	**Domestic**	94
	**Wildlife**	1630
**Vietnam**	**Domestic**	488
	**Wildlife**	3984

Table S 6: Number of wild and domestic animals sampled for detection of viruses in 34 countries.

Virus Family / Taxonomic unit	PCR tests: protocols used	Number of Tests
**Adenoviridae**	**Casas, Hexon gene**	2875
	**Wellehan et al, Pol gene**	6632
	**other-Casas, Hexon gene**	88
	**other-Felix Dressler, Bonn Institute Protocol**	13
	**other-Heim et al., 2003**	262
	**other-Wellehan et al, Pol gene**	88
**Arenaviridae**	**Lonzano et al, S gene**	26390
	**Unpublished UCD**	2159
	**other-**	833
	**other-Drosten et al., 2007**	191
	**other-Eric Leroy, CIRMF Protocol**	12
	**other-UCD unpublished**	48
	**other-Vieth et al., 2007 (Modified)**	2527
	**other-Vieth et al., L-gene**	2558
**Astroviridae**	**Atkins et al, RdRp gene**	6255
	**Chu et al, RdRp gene**	10763
	**other**	130
	**other-Atkins et al, RdRp gene**	88
	**other-Chu et al, RdRp gene**	88
	**other-Felix Dressler, Bonn Institute Protocol**	6
	**other-Logan et al., 2007**	262
**Bunyavirales**	**Briese et al, L gene**	14390
	**Briese et al, M gene**	9122
	**Briese et al, S gene**	10605
	**other-**	1276
	**other-Briese et al, S, M and L segments**	88
	**other-UCD unpublished**	51
**Caliciviridae**	**Reid et al, Capsid**	458
	**other-Felix Dressler, Bonn Institute Protocol**	6
	**other-Hoehne et al., 2006**	262
	**other-Logan et al., 2007**	262
**Coronaviridae**	**Ge et al., RdRp**	153
	**Modified Watanabe et al, RdRp gene**	133872
	**Quan et al, RdRp gene**	134105
	**Quan	Watanabe**	60
	**Tong S, et al. EID 2009, RdRp gene**	120
	**other**	167
	**other-**	1630
	**other-CDC**	822
	**other-Dare et al., 2007**	3359
	**other-Felix Dressler, Bonn Institute Protocol**	10

	**other-de Souza Luna et al, 2007, RdRp gene**	1483
**Filoviridae**	**Goldstein**	27136
	**Jaaskelainen et al, L gene**	33208
	**Modified Zhai et al, L gene**	70
	**Modified Zhai et al, L gene, Filo A**	8142
	**Modified Zhai et al, L gene, Filo A | B | C | D**	7710
	**Modified Zhai et al, L gene, Filo B**	8115
	**Modified Zhai et al, L gene, Filo C**	7999
	**Modified Zhai et al, L gene, Filo D**	8003
	**Modified Zhai et al, L gene, Filo MOD**	16049
	**Towner et al, NP gene**	41929
	**Unpublished CII**	35711
	**Unpublished, UCD**	154833
	**other**	417
	**other-**	1615
	**other-Eric Leroy, CIRMF Protocol**	12
	**other-Grard et al., 2011**	1719
	**other-Grolla, PHAC**	3
	**other-New filo**	51
	**other-Public Health Agency Canada**	556
	**other-Towner et al., 2006**	1719
**Flaviviridae**	**Modified Moureau et al, NS5 gene**	28326
	**Moureau et al, NS5 gene**	86391
	**Sanchez-Seco et al, NS5 gene**	5045
	**Unpublished UCD, NS5 gene**	1295
	**other-**	1556
	**other-Eric Leroy, CIRMF Protocol**	12
	**other-Lanciotti et al 1992**	1
	**other-Lanciotti_West Nile virus**	151
	**other-Linssen, 2000**	1
	**other-Makuwa, M., Souquire, S., Clifford, S. L., Mouinga-Ondeme, A., Bawe-Johnson, M., Wickings, E. J., Latour, S., Simon, F. & Roques, P. (2005). Identification of hepatitis B virus genome in faecal sample from wild living chimpanzee (Pan troglodytes troglodytes) in Gabon. J Clin Virol 34, S83-S88.**	156
	**other-Peter Simmonds protocol**	555
	**other-Sharp/Simmonds**	1
**Hantaviridae**	**Aitichou et al (2005)**	147
	**Klempa et al, L-Segment**	5684
	**Klempa et al, N gene**	7479
	**Modified Aitichou et al, S gene**	16111

	**Modified Raboni et al, S gene**	8061
	**Modified Raboni et al, S segment**	1999
	**Modified Zhai et al, L gene, Filo D**	2
	**other**	17
	**other-**	1224
	**other-Araujo et al., 2011**	108
	**other-PCR-ICB, S gene**	68
	**other-qPCR-ICB, S gene**	74
**Hepadnairidae**	**other-Hepatitis A virus**	38
	**other-Hepatitis B virus**	38
**Herpesviridae**	**Chmielewicz et al, Term gene**	6232
	**Van DeVanter et al, Pol gene**	8290
	**other-**	773
	**other-Garner et al. 2009**	30
	**other-Herpes B**	828
	**other-Latimer et al. 2011**	90
**Orthomyxoviridae**	**Anthony et al, M gene**	115476
	**Liang et al, PB1 gene**	5278
	**Liang unpublished CII, PB1 gene**	110346
	**Unpublished CII, Influenza A, PB1 gene**	2458
	**other-**	2094
	**other-Gerloff 2016**	4890
	**other-Liang**	51
	**other-Liang et al, PB1 gene**	87
	**other-Liang unpublished CII, PB1 gene**	573
	**other-Protocol provided by Laboratory at National Institute of Infectious Diseases (NIID)**	4189
	**other-Unpublished CII, Influenza A, PB1 gene**	12
	**other-WHO-Avian Influenza A**	6
	**other-Wang, W., et al. 2009**	162
**Papillomaviridae**	**Forslund et al, L1 gene**	558
**Paramyxoviridae**	**Modified Feldman et al, L gene**	12582
	**Tong et al, PAR**	110940
	**Tong et al, Pol gene**	31542
	**Tong et al, RES**	5575
	**Wacharapluesadee et al, N gene**	5214
	**Wacharapluesadee et all, N gene**	587
	**other**	167
	**other-**	1371
	**other-Eric Leroy, CIRMF Protocol**	10
	**other-Gerloff 2016**	331

	**other-Nested conventional PCR targeting on the L-gene**	88
	**other-Protocol of HUA National Key Laboratory of Veterinary Biotechnology**	46
	**other-Tong, S et al, (2008)**	306
	**other-Unpublished methodology**	2324
	**other-adapted from Feldman et al., 2009**	822
**Parvoviridae**	**Kapoor et al, NS1 gene**	5001
	**other-Kapoor et al, NS1 gene**	88
	**other-Peter Simmonds protocol**	212
	**other-Sharp CP, LeBreton M, Kantola K, Nana A, Diffo Jle D, Djoko CF, Tamoufe U, Kiyang JA, Babila TG, Ngole EM, Pybus OG, Delwart E, Delaporte E, Peeters M, Soderlund-Venermo M, Hedman K, Wolfe ND, Simmonds P. (2010) Widespread infection with homologues of human parvoviruses B19, PARV4, and human bocavirus of chimpanzees and gorillas in the wild. Journal of Virology 84(19):10289-96.**	490
**Peribunyaviridae**	**Briese et al, L segment**	439
	**Unpublished UCD, L segment**	7275
**Phenuiviridae**	**Modified Sanchez-Seco et al, L gene**	1320
	**other-Drosten et al., 2002**	370
	**other-Modified Sanchez-Seco et al, L gene**	88
**Picornaviridae**	**Nix et al, VP3/VP1**	1401
	**Unpublished CII, 5 UTR**	4470
	**Unpublished CII, UTR gene**	2
	**Unpublished CII, VP4/2 gene**	1289
	**other-Brever et al, VP2 gene**	3
	**other-Dierssen et al., 2007**	262
	**other-Encephalomyocarditis virus**	38
	**other-Felix Dressler, Bonn Institute Protocol**	25
	**other-Nix W.A. (2006), VP1 gene**	4
	**other-Nix W.A. 2006, VP1 gene**	10
	**other-Nix et al, VP3/VP1**	91
	**other-Nix, W.A. 2006**	6
	**other-Unpublished CII**	210
	**other-Unpublished CII, 5 UTR**	88
	**other-Unpublished CII, VP4/2 gene**	88
**Polyomaviridae**	**Johne, VP1 gene**	5345
	**other-Johne, VP1 gene**	88
**Poxviridae**	**Bracht et al, Pol and Topoisomerase genes**	370
	**Bracht et al, Topoisomerase gene**	1876

	**Bracht et al.,Pol gene**	963
	**Inoshima et al, B2L gene**	3453
	**Nitsche et al, RdRp gene**	6660
	**Nollens et al, p42K gene**	95
	**other-Eric Leroy, CIRMF Protocol**	10
	**other-Inoshima et al, B2L gene**	88
	**other-Yu Li et al**	415
**Reoviridae**	**Palacios et al, VP1 gene**	1684
	**Unpublished CII, VP1 gene**	9126
	**other-**	750
	**other-Bourhy, H. et al**	519
	**other-Felix Dressler, Bonn Institute Protocol**	6
	**other-Hoehne et al., 2006**	262
	**other-Jothikumar, N., Kang, G., & Hill, V. R. (2009)**	478
	**other-Palacios et al, VP1 gene**	88
	**other-Unpublished CII, VP1 gene**	88
	**other-Wellehan JF et al. , RdRp gene**	61
**Retroviridae**	**Courgnaud et al, Pol gene**	3221
	**Goldberg et al, LTR**	1409
	**Goldberg et al, Pol gene**	2562
	**other-**	390
	**other-Clewley et al, RdRp gene**	458
	**other-Courgnaud et al, Pol gene**	88
	**other-Duong et al**	84
	**other-Goldberg et al, Pol gene**	88
	**other-Vallejo et al, tax gene**	7
**Rhabdoviridae**	**Modified Vazquez-Moron et al, N gene**	1558
	**Unpublished CII, L pol gene**	21773
	**other-**	1084
	**other-Bourhy, H. et al**	1981
	**other-Grard, Plos Pathogens 2012, L gene**	1198
	**other-Institute Pasteur Paris, pol gene**	6869
	**other-Modified Vazquez-Moron et al, N gene**	88
	**other-Towner et al., 2006**	1641
	**other-Unpublished methodology**	419
**Togaviridae**	**Modified Sanchez-Seco et al, NSP4 gene**	7583
	**Sanchez-Seco et al, NS5 gene**	1
	**Sanchez-Seco et al, NSP4 gene**	2442
	**other-**	937
	**other-Eric Leroy, CIRMF Protocol**	12
	**other-Grywna et al., 2010**	3770
	**other-Lanciotti, 2000**	1

	**other-Linssen 2000_Eastern equine encephalitis virus**	151
	**other-Linssen 2000_Venezuelan equine encephalitis virus**	151
	**other-Linssen 2000_Western equine encephalitis virus**	151
	**other-PREDICT P-011**	64

Table S 7: Number of tests performed using various testing protocols of Conventional PCR, Conventional RT-PCR, Real-time PCR, and Next Generation Sequencing for detection of viruses in wildlife and domestic animals.

	**PubMed Search (Log)**		
Predictors	*Estimates*	*CI*	*p*
(Intercept)	-2.79	-4.05 – -1.53	**<0.001**
Adenoviridae	*Reference*		
Arenaviridae	-0.18	-1.74 – 1.38	0.821
Arteriviridae	4.46	1.10 – 7.82	**0.009**
Asfarviridae	3.72	-1.86 – 9.30	0.191
Astroviridae	2.30	-0.67 – 5.26	0.129
Caliciviridae	0.82	-1.05 – 2.70	0.390
Circoviridae	2.40	-1.63 – 6.43	0.244
Coronaviridae	2.98	1.04 – 4.91	**0.003**
Filoviridae	3.80	1.78 – 5.82	**<0.001**
Flaviviridae	2.43	1.05 – 3.81	**0.001**
Hantaviridae	-1.18	-2.84 – 0.48	0.163
Hepadnaviridae	4.10	1.13 – 7.07	**0.007**
Herpesviridae	1.08	-0.31 – 2.46	0.128
Nairoviridae	2.23	-0.48 – 4.94	0.106
Orthomyxoviridae	3.02	0.48 – 5.55	**0.020**
Papillomaviridae	1.15	-0.32 – 2.61	0.125

Paramyxoviridae	3.76	2.03 – 5.49	**<0.001**
Parvoviridae	3.49	1.68 – 5.29	**<0.001**
Peribunyaviridae	2.42	0.91 – 3.93	**0.002**
Phenuiviridae	3.88	1.50 – 6.25	**0.001**
Picobirnaviridae	1.24	-4.33 – 6.80	0.663
Picornaviridae	1.82	0.13 – 3.51	**0.035**
Pneumoviridae	3.35	0.37 – 6.33	**0.028**
Polyomaviridae	3.85	1.58 – 6.11	**0.001**
Poxviridae	3.89	2.28 – 5.50	**<0.001**
Reoviridae	1.83	0.15 – 3.51	**0.033**
Retroviridae	2.89	1.29 – 4.50	**<0.001**
Rhabdoviridae	2.73	1.17 – 4.29	**0.001**
Tobaniviridae	2.91	-0.45 – 6.26	0.090
Togaviridae	3.77	2.15 – 5.40	**<0.001**
GenBank Hits (Log)	0.72	0.61 – 0.82	**<0.001**
Observations	530		

Table S 8: Gaussian Generalized linear mixed model to understand association between virus family and PubMed Hists on the PubMed Search hits.
PubMed Search (Log)**~ Virus Family + PubMed hits, family = Gaussian**

PREDICT Consortium: Ola Ababneh, Mustafa Ababneh, Jum Rafiah Abd Sukor, Mohd Lutfi Abdullah, Josefina Abedin, Ehab Abu-Basha, Mohamed Ahmed Ali, Junior Beal Akoundze, Joël Akpaki, Sief Addeen Al Hanandeh, Bilal Al Omari, Abdullah Al Shakil, Mohammad Borhan Al-Zghoul, Stephenie Ann Albart, Abdullah Alshammari, Basil H. Amarneh, William Ampofo, Victoria Andrew, Dao Le Anh, Ulaankhuu Ankhanbaatar, Simon Anthony, Ungke Antonjaya, Kidan Araya, Jallah Arku, Norsharina Arshat, Theodore Asigbee, Ohnmar Aung, Joseph Awuni, James Ayukebong, Mohamed Saifullah Mohd Azian, Nor Adilah Aziz, Aminata Ba, Ganzorig Baasan, Zena Babu, Ola Bagato, Aboubacar Bamba, Djeneba Bamba, James Bangura, Ariunbaatar Barkhasbaatar, June Barrera, Cale Basaraba, Samuel Bel-Nono, Manjunatha Belaganahalli, Desalegn Belay, Jaber Belkhiria, Ridzki M.F. Binol, Brian Bird, Manisha Bista, Pitu Biswas, Matthew Blake, Linda Boatemaa, Margret Bonason, James Brandful, Joseph Brown, John Brownstein, Alpha Oumar Camara, Mamadi Camara, Salif Camara, Daniel Chai, Debapriyo Chakraborty, Hannah Chale, Ashok Chaudhary, Sokha Chea, Aleksei A. Chmura, Andrew Chow, Carolina Churchill, Abraham Commey, Emmanuel Couacy-Hymann, Julien Kalpy Coulibaly, Michael Cranfield, Wirda Damanik, Batchuluun Damdinjav, Norhidayah Danial, Peter Daszak, Runie David, Patrick Dawson, Ardjouma Dembele, Awa Deme, James Desmond, Aghnianditya Kresno Dewantari, Jasjeet Dhanota, Tapan Dhole, Nguyen Thi Diep, Aristide Dionkounda, Gaye Laye Diop, Kimberly Dodd, Otilia Dogbey, Tumendemberel Dorjnyam, Mireille Dosso, Kalil Doumbouya, Mohamed Idriss Doumbouya, Megan Doyle, Simone Dramou, Tracy Drazenovich, Dang Duc Anh, Bach Duc Luu, Prateep Duengkae, Vu Trong Duoc, Tran Nhu Duong, Veasna Duong, Huda Dursman, Philippe Dussart, Shusmita Dutta, Tan Jun Ee, Abel Ekiri, Amira S. El Rifay, Rabeh El Shesheny, Ahmed N. El Taweel, Zena Emmanuel, Jonathan H. Epstein, Jason Euren, Tierra Smiley Evans, Alaa Fahmawi, Simeon Fahn, Yasha Feferholtz, Jinnat Ferdous, Amanda Fine, Meerjady Sabrina Flora, Leilani Francisco, Lem Fui Fui, Taylor Gabourie, Millawati Gani, Michael Garbo, Nicole Gardner, Aiah Gbakima, Marie Pelagie Atrou Gbamele, Xingyi Ge, Lee Heng Gee, Brooke Genovese, Alexandra Gibson, Kirsten Gilardi, Martin Gilbert, Amethyst Gillis, Andrew Ginsos, Privat Godji Gnabro, Tracey Goldstein, Mokhtar R. Gomaa, Jules Gomis, Kevin Gonzalez, Zoe Grange, Denise Greig, Michael Grodus, Kpon Kakeuma Romeo Gueu, Leticia Gutierrez, Dan Marcelin Haba, Emily Hagan, Abdul Hai, Suraya Hamid, Daniel K. Harris, Abdul Kadir Abu Hashim, Moushumi Hassan, Quaza Nizamuddin Hassan, Qun He, Thiravat Hemachudha, Helena Henry, Ronald Herbert, Zaidoun Hijazeen, Moukala Ndolo Hilarion, Rebecca Hill, Nguyen Thi Hoa, Paul Horwood, Md. Enayet Hossain, Saddam Hossain, Moh Moh Htun, Ben Hu, Tom Hughes, Vibol Hul, Vo Van Hung, Fatima Hussein, Ghislain Dzeret Indolo, Diah Iskandriati, Ariful Islam, Md. Tarikul Islam, Shariful Islam, Mohd Isnaim Ismail, Zuhair Bani Ismail, Jacques Iyanya, Joel Judson Jaimin, Amara Jambai, Jeffrine Rovie Ryan Japning, Alexter Japrin, Frantz Jean Louis, Titus Joe, Christine K. Johnson, Erica Johnson, Damien Joly, Jyotsna Joshi, Jusuf Kalengkongan, Douokoro Kalivogui, Nenneh Kamara-Chieyoe, Joseph Kamau, Eddy Kambale Syaluha, Ahmed Kandeil, Yagouba Kane, William Karesh, Kandeh Kargbo, Dibesh Karmacharya, Novie Kasenda, Ghazi Kayali, Ahmed S. Kayed, Rudovick Kazwala, Changwen Ke, Lucy Keatts, Nigatu Kebede, Nigatu Kebede, Bouaphanh Khamphaphongphane, Chong Chee Kheong, Christopher Kilonzo, Ma-Sue Koffa, Amos G. Kollie, Marcel Sidiki Kondiano, Michel Koropo, Valere Kouamé Kouakou, Eugene Kouassi Koffi, Mariam Kourouma, Abdoulaye Ousmane Koutate, Citra Livi Kowel, Hermann Assemien Krou, Charles Kumakamba, Tina Kusumaningrum, Omnia Kutkat, François Lamah, Nguyen Thi Lan, Jennifer Lane, Christian Lange, Emmanuel Larmouth, Alice Latinne, Anne Laudisoit, Joseph Diffo Le

Doux, Elizabeth Leasure, Katherine Leasure, Mat LeBreton, Jimmy Lee, Helen Lee, Mei Ho Lee, Amara Leno, Hongying Li, Eliza Liang, Neal Liang, Dorothy Lim, W. Ian Lipkin, Jun Liu, Modou Moustafa Lo, Leonoris Lojivis, Nguyen Van Long, Nguyen Van Long, Ashley Lucas, Jean Paul Lukusa, Victor Lungay, Shongo Lushima, Julius Lutwama, Wenjun Ma, Catherine Machalaba, Grace Mwangoka, Walter Simon Magesa, Sara H. Mahmoud, Maria Makuwa, Asha Makweta, Abdullah Al Mamun, Prajwol Manandhar, Patarapol Maneeorn, Harjeet Mann, Pe Bhele Maomy, Victorine Maptue, Stephanie Martinez, Alice Mathew, Yanne Vanessa Mavoungou, Min Thein Maw, Jonna Mazet, Placide Mbala, Emmanuel Mbuba, Eric Mbunwe, David McIver, Emma Mendelsohn, Valchy Bel-Bebi Miegakanda, Maureen Miller, Phan Quang Minh, Happy Mkali, Yassmin Moatasim, Jean Vivien Mombouli, Corina Monagin, Diego Montecino-Latorre, Arsene Mossoun Mossoun, Ahmed Mostafa, Moctar Mouiche, Romain Bagamboula Mpassi, Alphonce Msigwa, Antoine Mudakikwa, Laura Benedict Mugok, Prime Mulembakani, Suzan Murray, Fakhrul Hatta Musa, Pacifique Musabimana, Samson Mutura, Tunu Mwamlima, Mwokozi Mwanzalila, Tin Tin Myaing, Theingi Win Myat, Aung Myo Chit, Magassouba N'faly, Manzan Jean N'Guettia, Anatole N'télo, Sylivia Nakimera, Vu Sinh Nam, Rajindra Napit, Senthilvel K.S.S. Nathan, Isamara Navarrete-Macias, Kortu M. Ndebe, Amadou Ndiaye, Daouda Ndiaye, Yohannes Negash, Nguyen Thi Thanh Nga, Ipos Ngay, Pham Thi Bich Ngoc, Fabien Niama, Rock Aimé Nina, Schadrack Niyonzima, Felix Nkom, Cynthia Nkoua, Noorliza Noordin, Rachmitasari Noviana, Julius Nwobegahay, Julius Nziza, Daniel O'Rourke, Tammie O'Rourke, Evangeline Obodai, Ricky Okello Okwir, Kevin Olival, Sarah Olson, Onkirotin Dionne Olva, Victoria Ontiveros, Fernandes Opook, Joko Pamungkas, Chandrawathani Panchadcharam, Pranav Pandit, Henri-Joseph Parra, Tran Minh Phuc, Nguyen Thanh Phuong, Jackson Y. Poultolnor, Saman Pradhan, Eunah Cho Preston, Mathieu Pruvot, Dulam Purevtseren, Dhiraj Puri, Le Tin Vinh Quang, Novie Rachmitasari, Kaisar Rahman, Mizanur Rahman, Mohammed Ziaur Rahman, Mustafizur Rahman, Diana Ramirez, Nistara Randhawa, Samita Raut, Joseph Rosario, Albert Ross, Noam Ross, Melinda Rostal, Pamela Roualdes, Eddy Rubin, Aftab Uddin Rumi, Christina Rundi, Melkor Sackie, Dodi Safari, Zikankuba Sijali, Sandra G. Samuels, Mathias Sango, Ammar Rafidah Saptu, Suryo Saputro, Daniel N'guessan Saraka, Alvis A. Sartee, Sia Alida Sayandouno, Karen Saylors, Mame Cheikh Seck, Victoria Sedor, Shahanaj Shano, Ajay Narayan Sharma, Velsri Sharminie, Mahmoud M. Shehata, Gafur Sheikh, Zhengli Shi, Enkhtuvshin Shiilegdamba, Bishwo Shrestha, Rima Shrestha, Mohammed Sidibey, Soubanh Silithammavong, Daniel Simon, Emilly Sion, Symphorosa Sipangkui, Frankie Thomas Sitam, Brett Smith, Bridgette Smith, Woutrina Smith, Batsikhan Sodnom, Benard Ssebide, Maria Suleiman, Ava Sullivan, Nur Amirah Md Sungif, Richard Suu-Ire, Mouhamed Sy, Jean Michel Takuo, Hani Talafha, Ubald Tamoufe, Emmanuel Tetteh, Aung Than Toe, Lanash Thanda, Ngo Thanh Long, Wai Zin Thein, Watthana Theppangna, Nguyen Duc Thinh, Hoang Bich Thuy, Nguyen Thu Thuy, Eri Togami, Moise Bendoua Tolno, Kevin Tolovou, Rahmat Topani, Alexandre Tremeau-Bravard, Ian Trupin, Jean Claude Tumushime, Kyaw Yan Naing Tun, Joseph Turay, Helal Uddin, Marcela Uhart, Nicole Ureda, Marc Valitutto, Khebir Verasahib, Megan Vodzak, Supaporn Wacharapluesadee, Mohammad Yuery Wazlan Abdul Wahad, Brooke Watson, Heather Wells, Allison White, Anna Willoughby, Ageng Wiyatno, David Wolking, Xinglou Yang, Lim Ming Yao, Sayon Yombouno, Cristin Young, Carlos Zambrana-Torrelio, Zahidah Izzati Zeid, Ghadeer Zghoul, Libiao Zhang, Yunzhi Zhang, Guangjian Zhu, Dawn Zimmerman, and Daba Zoumarou.

PREDICT-1 (2009-2014)

Alonso Aguirre, Luis Aguirre, Mark-Joel Akongo, Erika Alandia Robles , Laurentius Ambu, Simon Anthony, Ungke Antonjaya, Glenda Ayala Aguilar, Luis Barcena, Rosario Barradas, Misliah Mohamad Basir, Tiffany Bogich, Gerard Bounga, John Brownstein, Philippe Buchy, David Bunn, Denis Byaruba, Ken Cameron, Dennis Carroll, Nancy Cavero, Manuel Cespedes, Xiaoyu Che, Sokha Chea, Charles Chiu, Aleksei Chmura, Kimashalen Chor, Andrew Clements, Michael Cranfield, Luz Dary Acevedo, Peter Daszak, Runie David, Angelica de Almeida Campos, Micaela De La Puente, Xavier de Lamballerie, Catia de Paula, Eric Delwart, Joseph Diffo Le Doux, Catherine Doyle-Capitman, Prateep Duengkae, Edison Durigon, Jonathan H. Epstein, Joseph Fair, José R. Ferrer-Paris, Amanda Fine, Pierre Formenty, Isabel Galarza, Joel Garcia, Kirsten Gilardi, Martin Gilbert, Amethyst Gillis, Andrew Ginsos, Tracey Goldstein, Benoit Goossens, Gilda Grard, Zoe Greatorex, Denise Greig, Emily Hagan, Laurie Harris, Qun He, Thiravat Hemachudha, Peta Hitchens, Parviez Hosseini, Tom Hughes, Samath In, Volga Iñíguez, Diah Iskandriati, Ariful Islam, Jacques Iyanya, Komal Jain, Abd. Aziz Jamaluddin, Jeffrine Rovie Ryan Japning, Christine Johnson, Damien Joly, Kate Jones, Priscilla Joyner, Serge Kaba, Eddy Kambale, William Karesh, Dibesh Karmacharya, Abdulhameed Kataregga, Rudovick Kazwala, Changwen Ke, Terra Kelly, Chong Chee Kheong, Kongsy Khammavong, A. Marm Kilpatrick, Samsir Laimun, Mat LeBreton, Helen Lee, Mei Ho Lee, Jimmy Lee, Eric LeRoy, Jordan Levinson, Marc Levy, Eliza Liang, Neal Liang, Dorothy Lim, Rolando Limachi, W. Ian Lipkin, Elizabeth Loh, Leonoris Lojivis, Linda J. Lowenstine, José Luis Mollericona, Shongo Lushima, Wenjun Ma, Catherine Machalaba, Ruth Maganga, Maria Makuwa, Joseph Malakalinga, Patarapol Maneeorn, Melissa Manhas, Pete Marra, Alice Mathew, Jonna Mazet, Rachael Mbabazi, Placide Mbala, Rodrigo Medellín, Patricia Mendoza, Sireeda Miller, Flavia Miranda, Megan Mitchell, Ramlan Mohamed, Debbie Mollard, Corina Monagin, Stephen Morse, Wivine Mouellet, Isabel Moya, Antoine Mudakikwa, Laura Benedict Mugok, Prime Mulembakani, Yovanna Murillo, Kris Murray, Suzan Murray, Jean-Jacques Muyembe Tamfum, Sylivia Nakimera, Fernando Nassar, Senthilvel K.S.S. Nathan, Isamara Navarrete-Macias, Ipos Ngay, Schadrack Niyonzima, Felix Nkom, Noorliza Noordin, Rachmitasari Noviana, Olivier Nsengimana, Julius Nziza, Lucy Ogg Keatts, Rafael Ojeda-Flores, Ricky Okwir Okello, Kevin Olival, Sarah Olson, Alain Ondzie, Daniel O'Rourke, Tammie O'Rourke, Joko Pamungkas, Janusz Paweska, Alisa Pereira, Victoria Pereira, Alberto Perez, Jocelyn Perez, Simorn Phon, Jum Rafiah Abd Sukor, Diana Ramirez, Patricia Reed, Dan Rejmanek, Oscar Rico, Rosario Rivera, Monica Romero, Joseph Rosario, Melinda Rostal, Celina Roy, Christina Rundi, Uus Saepuloh, Dodi Safari, Zikankuba Sijali, Karen Saylors, Brad Schneider, Jessica Schwind, Zhengli Shi, Sinpakhome Singhalath, Symphorosa Sipangkui,Frankie Thomas Sitam, Tierra Smiley Evans, Brett Smith, Kristine Smith, Woutrina Smith, Benard Ssebide, Fabiola Suárez, Maria Suleiman, Gerardo Suzan, Jean Michel Takuo, Ubald Tamoufe, Lanash Thanda, Nguyen Thi Thanh Nga, Kate Thomas, Herminio Ticona, Alexandre Tremeau-Bravard, Marcela Uhart, Nguyen Van Long, Elizabeth VanWormer, Khebir Verasahib, Sandra Villar, Supaporn Wacharapluesadee, Wendy Weisman, Michael Westfall, Chris Whittier, Leanne Wicker, Ageng Wiyatno, Nathan Wolfe, David Wolking, Angela Yang, Carlos Zambrana-Torrelio, Carlos Zariquiey, Shu-Yi Zhang, Baudelaire Zorine Nkouantsi, and Zainal Zainuddin.
