# Supplementary material for: Predicting the potential for zoonotic transmission and host associations for novel viruses
Source: Commun Biol. 2022 Aug 19;5:844. doi: 10.1038/s42003-022-03797-9 (PMC9390964; doi:10.1038/s42003-022-03797-9)
Supplement: Supplementary file 2 — Description of Additional Supplementary Files [file 42003_2022_3797_MOESM2_ESM.pdf]

Predicting the potential for zoonotic transmission and host associations for novel viruses

Authors:

P. S. Pandit[1*], S.J. Anthony[2**], T. Goldstein[1**], K. J. Olival[3], M. M. Doyle[1], N. R. Gardner[1], B. Bird[1], W. Smith[1], D. Wolking[1], K. Gilardi[1], C. Monagin[1], T. Kelly[1], M. Uhart[1], J. H. Epstein[3], C. Machalaba[3], M. K. Rostal[3], P. Dawson[3], E. Hagan[3], A. Sullivan[3], H. Li[3], A. A. Chmura[3], A. Latinne[3], C. Lange[4], T. O'Rourke[4], S. Olson[5], L. Keatts[1], P. Mendoza[5], A. Perez[5], C. Dejuste de Paula[5], D. Zimmerman[6], M. Valitutto[6], M. LeBreton[7], D. McIver[8], A. Islam[3], V. Duong[9], M. Mouiche[7], Z. Shi[10], P. Mulembakani[11], C. Kumakamba[12], M. Ali[13], N. Kebede[14], U. Tamoufe[15], S. Bel-Nono[16], A. Camara[17], J. Pamungkas[18, 19], K. Coulibaly[20], E. Abu-Basha[21], J. Kamau[22], S. Silithammavong[8], J. Desmond[3], T. Hughes[3,23], E. Shiilegdamba[24], O. Aung[6], D. Karmacharya[25], J. Nziza[26], D. Ndiaye[27], A. Gbakima[28], Z. Sijali[29], S. Wacharapluesadee[30], E. Alandia Robles[31], B. Ssebide[26], G. Suzán[32], L. F. Aguirre[33], M. R. Solorio[34], T. N. Dhole[35], N. T. T. Nga[36], P. L. Hitchens[37], D. O. Joly[38], K. Saylors[4], A. Fine[5], S. Murray[7], W. Karesh[3], P. Daszak[3], J. A. K. Mazet[1], PREDICT Consortium, & C. K. Johnson[1*]

*Corresponding author: P.S. Pandit, pspandit@ucdavis.edu and C. K. Johnson, ckjohnson@ucdavis.edu

Description of Additional Supplementary Files

File name: Supplementary Data 1

Description: Species target list for novel viruses based on known hosts of virus to which novel viruses are predicted to generate links in the predicted network ($G_{predicted}$)

File name: Supplementary Data 2

Description: Data listing novel viruses from wildlife and their wildlife host. Data also describes the prioritized surveillance target wildlife hosts as predicted by network prediction models.

File name: Supplementary Data 3

Description: Data file listing available GenBank accession numbers for novel PREDICT viruses used in the study to generate network predictions.

File name: Supplementary Data 4

Description: FASTA file with nucleotide sequences for novel PREDICT viruses used in the study to generate network predictions for which GenBank accession numbers are not available currently.
